# Supplementary material for: Diversity of fish sound types in the Pearl River Estuary, China
Source: PeerJ. 2017 Oct 24;5:e3924. doi: 10.7717/peerj.3924 (PMC5659214; doi:10.7717/peerj.3924)
Supplement: Supplemental Information 1 [file peerj-05-3924-s001.zip › Supplemental figures/Supplemental Figure legends.docx]

**Fig. S1 Characteristic of the (A) 2 and (B) 1+1 call types.** Rows 1 and 2 are the oscillogram and sonogram, respectively, of a representative signal for each call type. Row 3 is the duration of a call as a function of the number of pulses within the call. Rows 4 is the pooled inter-pulsepeak interval of each pulse versus the order at which it occurs within a call. For the boxplot, the line inside the box indicates the median value, and the upper and lower box borders are the first and third quartiles, respectively. The length of the box is the interquartile range (IQR). The whiskers extend to the most extreme data within the limit of 1.5 IQRs from the end of the box. Open circles (o) denote mild outliers with values greater than 1.5 IQRs but fewer than 3 IQRs from the end of the box. Asterisks (*) denote extreme outliers with values greater than 3 box lengths from the upper or lower edges of the box. Sonogram configuration: FFT size, 96,000; window type, Hanning; overlap samples per frame, 95%.

**Fig. S2 Characteristic of the (A) 2+N_9_, (B) 2+N_10_ and (C) 2+N_18_ call types.** Rows 1 and 2 are the oscillogram and sonogram, respectively, of a representative signal for each call type. Row 3 is the duration of a call as a function of the number of pulses within the call. Rows 4 is the pooled inter-pulsepeak interval of each pulse versus the order at which it occurs within a call.

**Fig. S3 Characteristic of the (A) 3+N_9_, (B) 3+N_10_ and (C) 3+N_17_ call types.** Rows 1 and 2 are the oscillogram and sonogram, respectively, of a representative signal for each call type. Row 3 is the duration of a call as a function of the number of pulses within the call. Rows 4 is the pooled inter-pulsepeak interval of each pulse versus the order at which it occurs within a call.

**Fig. S4 Characteristic of the (A) 4+N_9_, (B) 4+N_10_ and (C)4+N_17_ call types.** Rows 1 and 2 are the oscillogram and sonogram, respectively, of a representative signal for each call type. Row 3 is the duration of a call as a function of the number of pulses within the call. Rows 4 is the pooled inter-pulsepeak interval of each pulse versus the order at which it occurs within a call.

**Fig. S5 Characteristic of the 5+N_10_ call type.** Rows 1 and 2 are the oscillogram and sonogram, respectively, of a representative signal for each call type. Row 3 is the duration of a call as a function of the number of pulses within the call. Rows 4 is the pooled inter-pulsepeak interval of each pulse versus the order at which it occurs within a call.

**Fig. S6 Characteristic of the (A) (1-)^2^+N_9_, (B) (1-)^2^+N_10_ and (C) (1-)^2^+N_12_ call type.** Rows 1 and 2 are the oscillogram and sonogram, respectively, of a representative signal for each call type. Row 3 is the duration of a call as a function of the number of pulses within the call. Rows 4 is the pooled inter-pulsepeak interval of each pulse versus the order at which it occurs within a call.

**Fig. S7 Characteristic of the (A) 1+2+N_10_ and (B) 1+2+N_18_ call types.** Rows 1 and 2 are the oscillogram and sonogram, respectively, of a representative signal for each call type. Row 3 is the duration of a call as a function of the number of pulses within the call. Rows 4 is the pooled inter-pulsepeak interval of each pulse versus the order at which it occurs within a call.

**Fig. S8 Characteristic of the (A) 2+1+N_9_ and (B) 2+1+N_10_ call types.** Rows 1 and 2 are the oscillogram and sonogram, respectively, of a representative signal for each call type. Row 3 is the duration of a call as a function of the number of pulses within the call. Rows 4 is the pooled inter-pulsepeak interval of each pulse versus the order at which it occurs within a call.

**Fig. S9 Characteristic of the (A) (2-)^2^+N_10_ and (B) 4+1+N_10_ call types.** Rows 1 and 2 are the oscillogram and sonogram, respectively, of a representative signal for each call type. Row 3 is the duration of a call as a function of the number of pulses within the call. Rows 4 is the pooled inter-pulsepeak interval of each pulse versus the order at which it occurs within a call.

**Fig. S10 Characteristic of the (A) 3+1+N_9_ and (B) 3+1+N_10_ call types.** Rows 1 and 2 are the oscillogram and sonogram, respectively, of a representative signal for each call type. Row 3 is the duration of a call as a function of the number of pulses within the call. Rows 4 is the pooled inter-pulsepeak interval of each pulse versus the order at which it occurs within a call.

**Fig. S11 Characteristic of the (A) 3+2+N_9_ and (B) 3+(1-)^2^+N_9_ call types.** Rows 1 and 2 are the oscillogram and sonogram, respectively, of a representative signal for each call type. Row 3 is the duration of a call as a function of the number of pulses within the call. Rows 4 is the pooled inter-pulsepeak interval of each pulse versus the order at which it occurs within a call.

**Fig. S12 Characteristic of the (A) (1-)^3^+N_9_, (B) (1-)^3^+N_10_ and (C) (1-)^3^+N_12_ call types.** Rows 1 and 2 are the oscillogram and sonogram, respectively, of a representative signal for each call type. Row 3 is the duration of a call as a function of the number of pulses within the call. Rows 4 is the pooled inter-pulsepeak interval of each pulse versus the order at which it occurs within a call.

**Fig. S13 Characteristic of the (A) (1-)^2^+2+N_9_ and (B) (1-)^2^+2+N_10_ call types.** Rows 1 and 2 are the oscillogram and sonogram, respectively, of a representative signal for each call type. Row 3 is the duration of a call as a function of the number of pulses within the call. Rows 4 is the pooled inter-pulsepeak interval of each pulse versus the order at which it occurs within a call.

**Fig. S14 Characteristic of the (1-)^2^+3+N_10_ call type.** Rows 1 and 2 are the oscillogram and sonogram, respectively, of a representative signal for each call type. Row 3 is the duration of a call as a function of the number of pulses within the call.

**Fig. S15 Characteristic of the (A) 2+(1-)^2^+N_9_ and (B) 2+(1-)^2^+N_10_ call types.** Rows 1 and 2 are the oscillogram and sonogram, respectively, of a representative signal for each call type. Row 3 is the duration of a call as a function of the number of pulses within the call. Rows 4 is the pooled inter-pulsepeak interval of each pulse versus the order at which it occurs within a call.

**Fig. S16 Characteristic of the (A) 2+1+2+N_9_ and (B) 2+1+2+N_10_ call types.** Rows 1 and 2 are the oscillogram and sonogram, respectively, of a representative signal for each call type. Row 3 is the duration of a call as a function of the number of pulses within the call. Rows 4 is the pooled inter-pulsepeak interval of each pulse versus the order at which it occurs within a call.

**Fig. S17 Characteristic of the (A) (1-)^4^+N_9_, (B) (1-)^4^+N_10_ and (C) (1-)^4^+N_12_ call types.** Rows 1 and 2 are the oscillogram and sonogram, respectively, of a representative signal for each call type. Row 3 is the duration of a call as a function of the number of pulses within the call. Rows 4 is the pooled inter-pulsepeak interval of each pulse versus the order at which it occurs within a call.

**Fig. S18 Characteristic of the (A) (1-)^3^+2+N_10_ and (B) (1-)^3^+3+N_10_ call types.** Rows 1 and 2 are the oscillogram and sonogram, respectively, of a representative signal for each call type. Row 3 is the duration of a call as a function of the number of pulses within the call. Rows 4 is the pooled inter-pulsepeak interval of each pulse versus the order at which it occurs within a call.

**Fig. S19 Characteristic of the (A) (1-)^2^+2+1+N_10_ and (B) (1-)^2^+2+3+N_10_ call types.** Rows 1 and 2 are the oscillogram and sonogram, respectively, of a representative signal for each call type. Row 3 is the duration of a call as a function of the number of pulses within the call.

**Fig. S20 Characteristic of the (A) 2+(1-)^3^+N_10_ and (B) 2+(1-)^4^+N_10_ call types.** Rows 1 and 2 are the oscillogram and sonogram, respectively, of a representative signal for each call type. Row 3 is the duration of a call as a function of the number of pulses within the call. Rows 4 is the pooled inter-pulsepeak interval of each pulse versus the order at which it occurs within a call.

**Fig. S21 Characteristic of the (A) (1-)^5^+N_9_ and (B) (1-)^5^+N_10_ call types.** Rows 1 and 2 are the oscillogram and sonogram, respectively, of a representative signal for each call type. Row 3 is the duration of a call as a function of the number of pulses within the call. Rows 4 is the pooled inter-pulsepeak interval of each pulse versus the order at which it occurs within a call.

**Fig. S22 Characteristic of the (A) (1-)^4^+2+N_10_ and (B) (1-)^4^+3+N_11_ call types.** Rows 1 and 2 are the oscillogram and sonogram, respectively, of a representative signal for each call type. Row 3 is the duration of a call as a function of the number of pulses within the call. Rows 4 is the pooled inter-pulsepeak interval of each pulse versus the order at which it occurs within a call.

**Fig. S23 Characteristic of the (A) (1-)^3^+2+1+N_10_ and (B) (1-)^4^+2+1+N_10_ call types.** Rows 1 and 2 are the oscillogram and sonogram, respectively, of a representative signal for each call type. Row 3 is the duration of a call as a function of the number of pulses within the call. Rows 4 is the pooled inter-pulsepeak interval of each pulse versus the order at which it occurs within a call.

**Fig. S24 Characteristic of the (A) (1-)^6^+N_10_ and (B) (1-)^7^+N_10_ call types.** Rows 1 and 2 are the oscillogram and sonogram, respectively, of a representative signal for each call type. Row 3 is the duration of a call as a function of the number of pulses within the call. Rows 4 is the pooled inter-pulsepeak interval of each pulse versus the order at which it occurs within a call.

**Fig. S25 Characteristic of the (A) (1-)^5^+2+N_10_ and (B) (1-)^5^+3+N_10_ call types.** Rows 1 and 2 are the oscillogram and sonogram, respectively, of a representative signal for each call type. Row 3 is the duration of a call as a function of the number of pulses within the call. Rows 4 is the pooled inter-pulsepeak interval of each pulse versus the order at which it occurs within a call.

**Fig. S26 Characteristic of the (A) (1-)^4^+(2-)^2^+N_10_ and (B) (1-)^5^+(2-)^2^+N_10_ call types.** Rows 1 and 2 are the oscillogram and sonogram, respectively, of a representative signal for each call type. Row 3 is the duration of a call as a function of the number of pulses within the call. Rows 4 is the pooled inter-pulsepeak interval of each pulse versus the order at which it occurs within a call.

**Fig. S27 Relative abundance of the 66 call types.**

**Fig. S28 Distribution pattern of the inter-pulspeak interval of each pulse versus the order at which it occurs within a call of all N_9_ and N_10_ call types.**
