# Supplementary figures and images for: Diversity of fish sound types in the Pearl River Estuary, China
Source: PeerJ. 2017 Oct 24;5:e3924. doi: 10.7717/peerj.3924 (PMC5659214; doi:10.7717/peerj.3924)

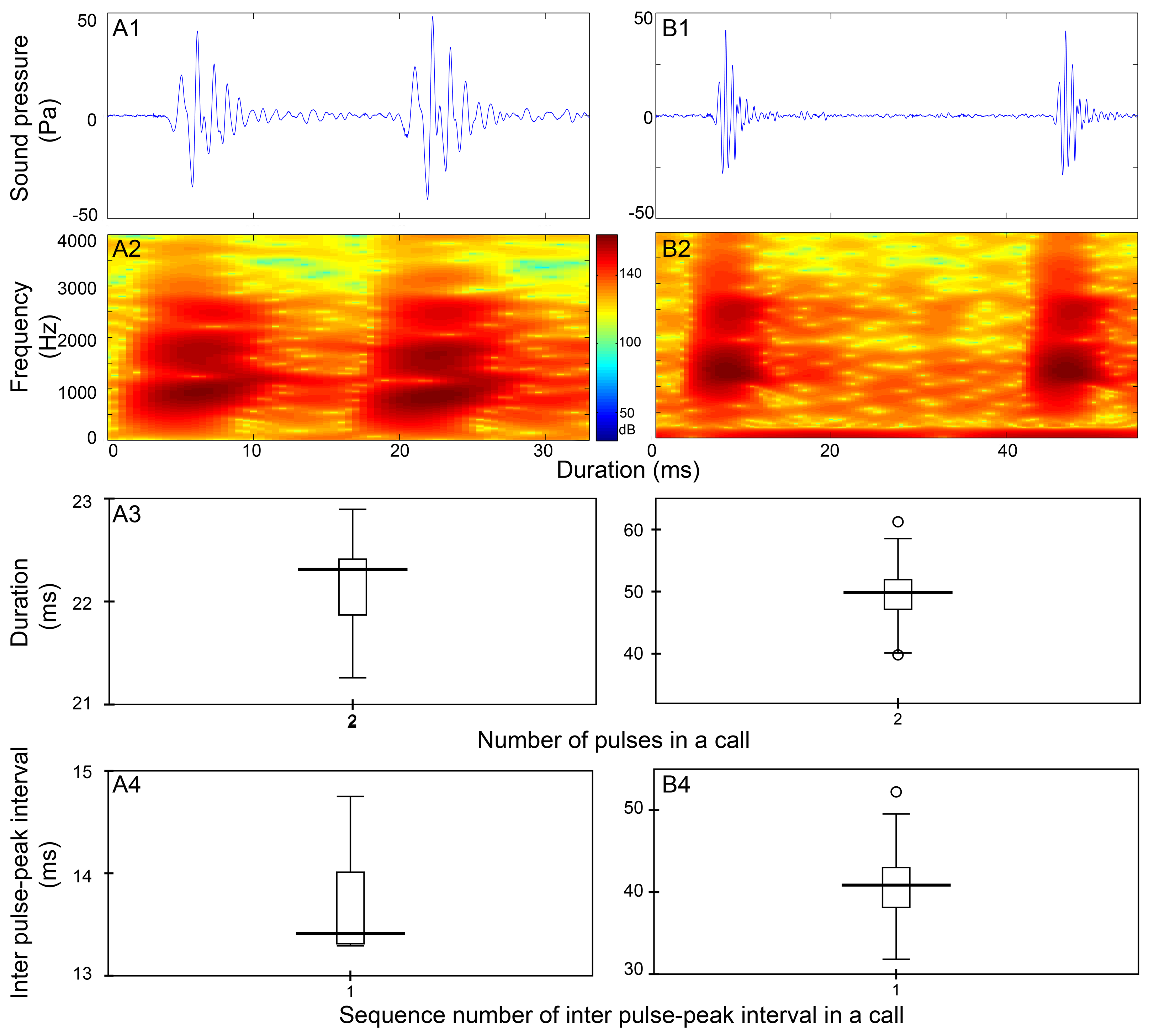

Supplement: Supplemental Information 1 [file peerj-05-3924-s001.zip › Supplemental figures/supplemental figures/Fig.S1.png]

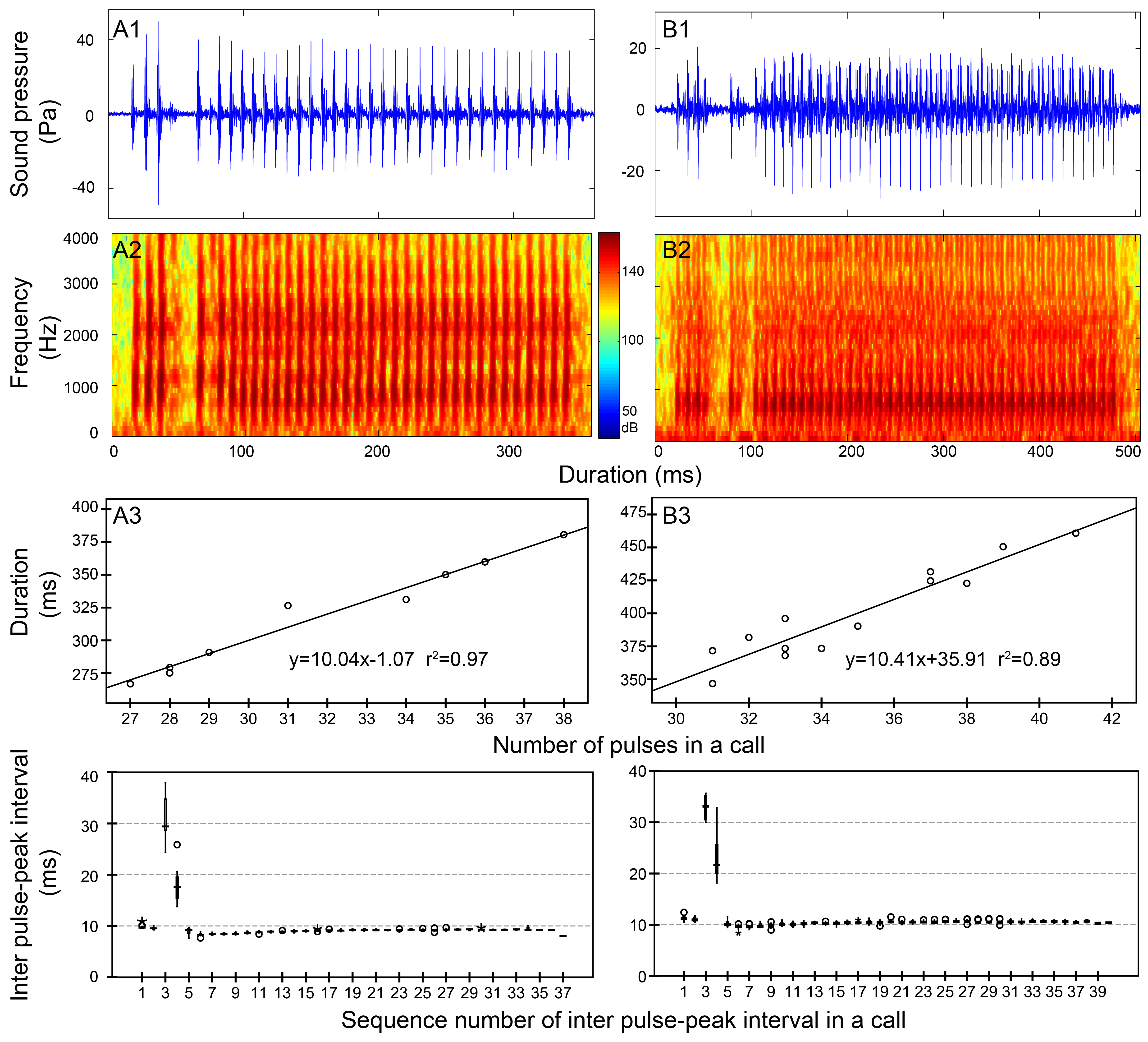

Supplement: Supplemental Information 1 [file peerj-05-3924-s001.zip › Supplemental figures/supplemental figures/Fig.S10.png]

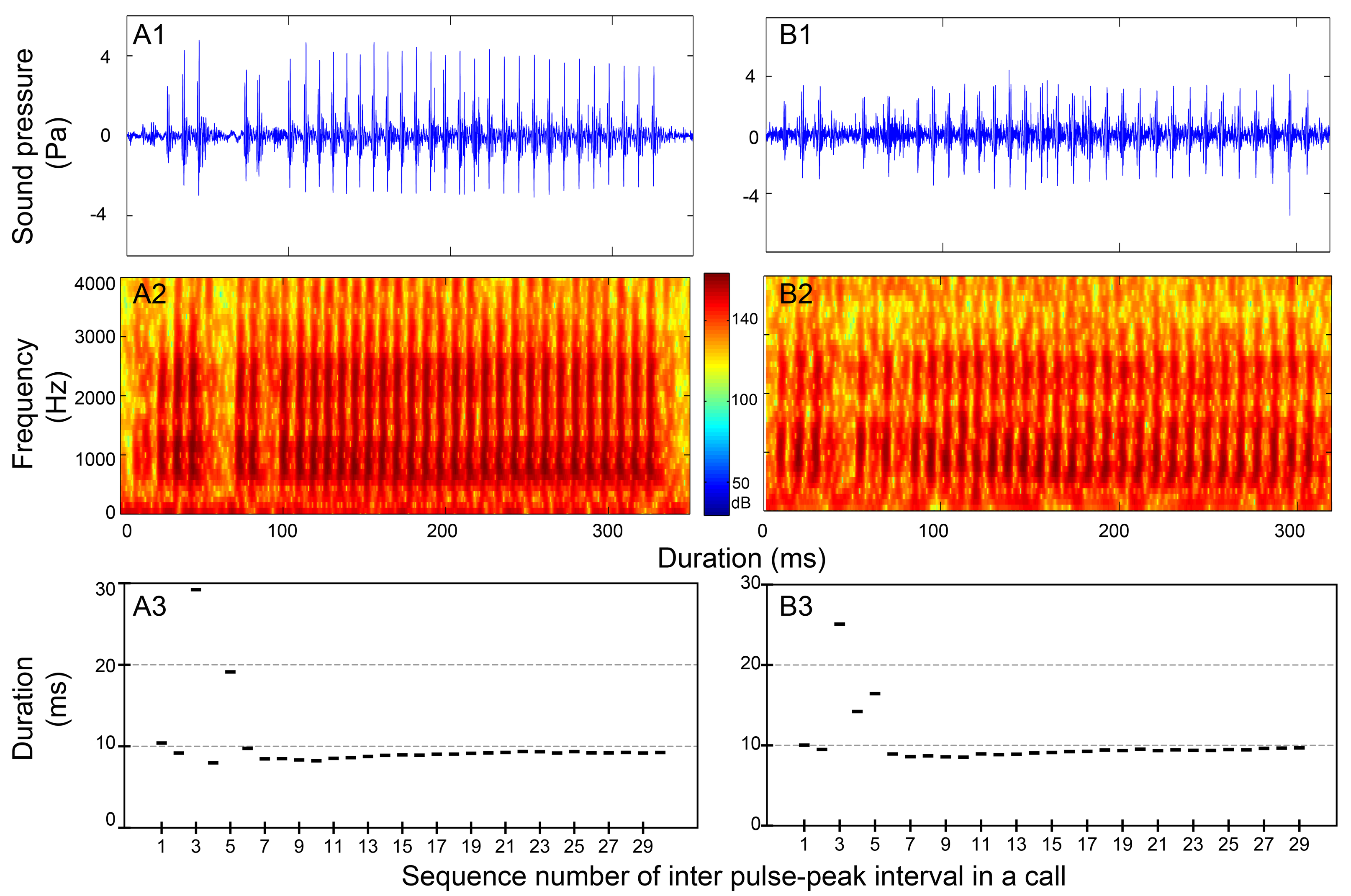

Supplement: Supplemental Information 1 [file peerj-05-3924-s001.zip › Supplemental figures/supplemental figures/Fig.S11.png]

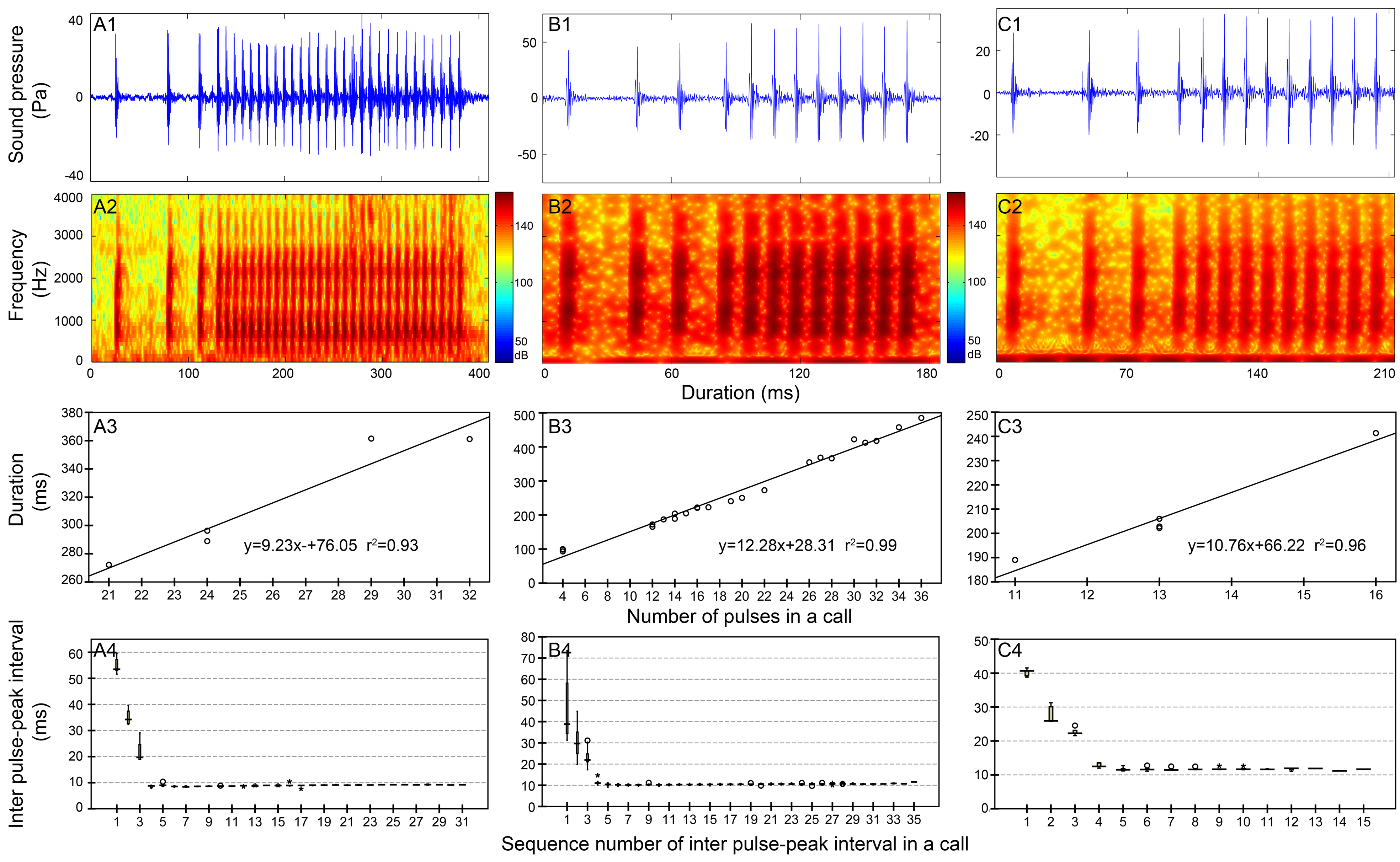

Supplement: Supplemental Information 1 [file peerj-05-3924-s001.zip › Supplemental figures/supplemental figures/Fig.S12.png]

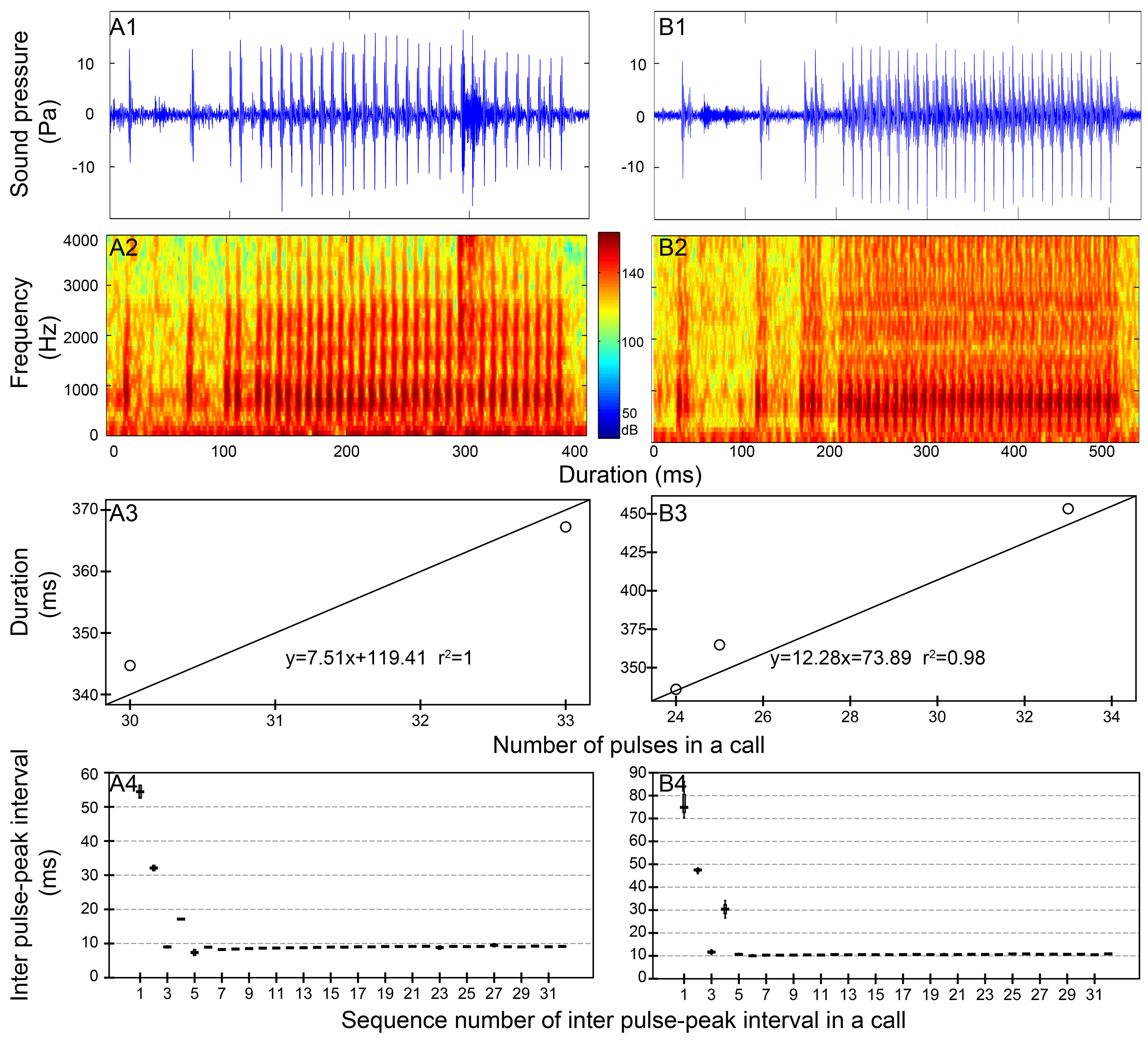

Supplement: Supplemental Information 1 [file peerj-05-3924-s001.zip › Supplemental figures/supplemental figures/Fig.S13.png]

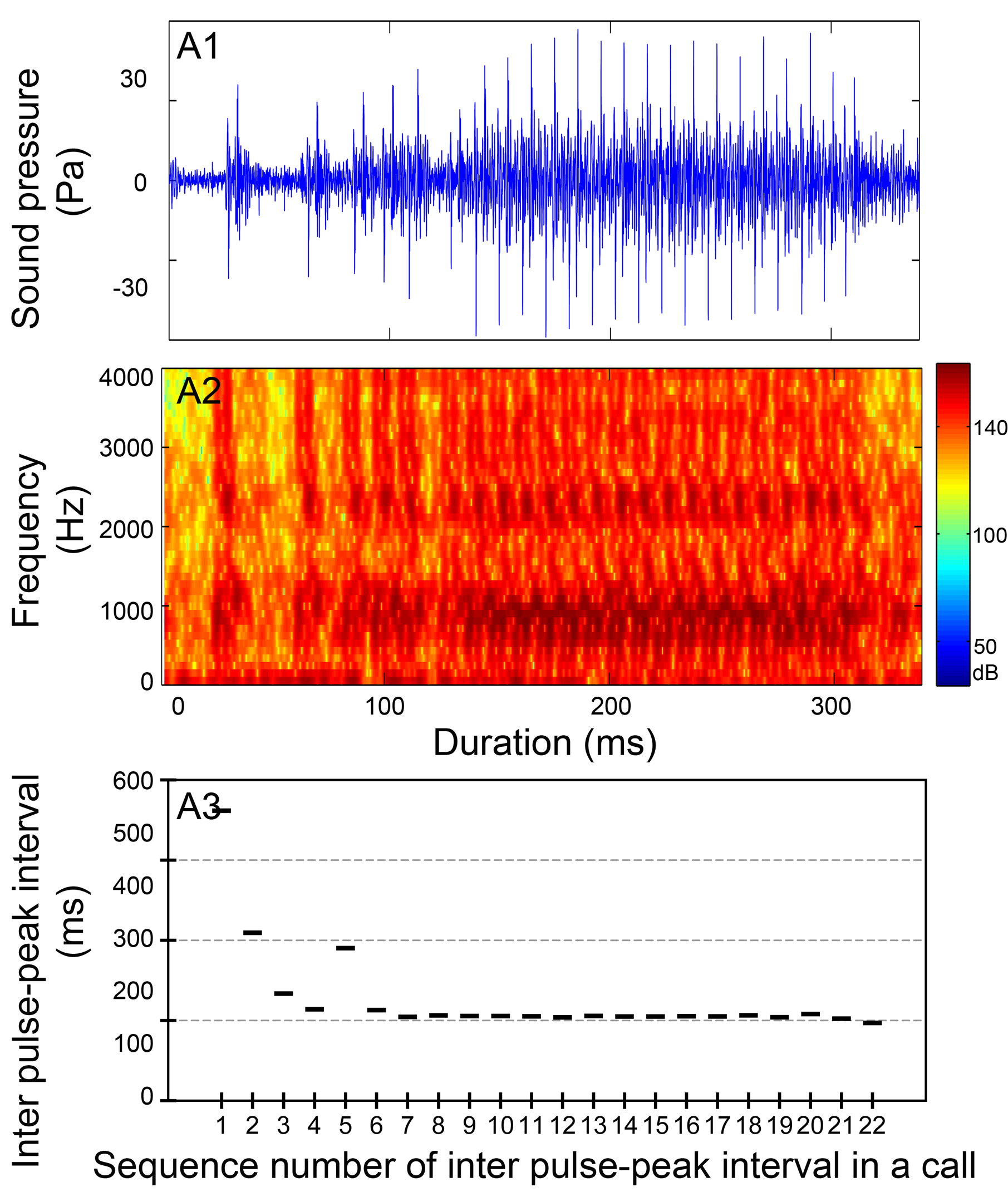

Supplement: Supplemental Information 1 [file peerj-05-3924-s001.zip › Supplemental figures/supplemental figures/Fig.S14 .png]

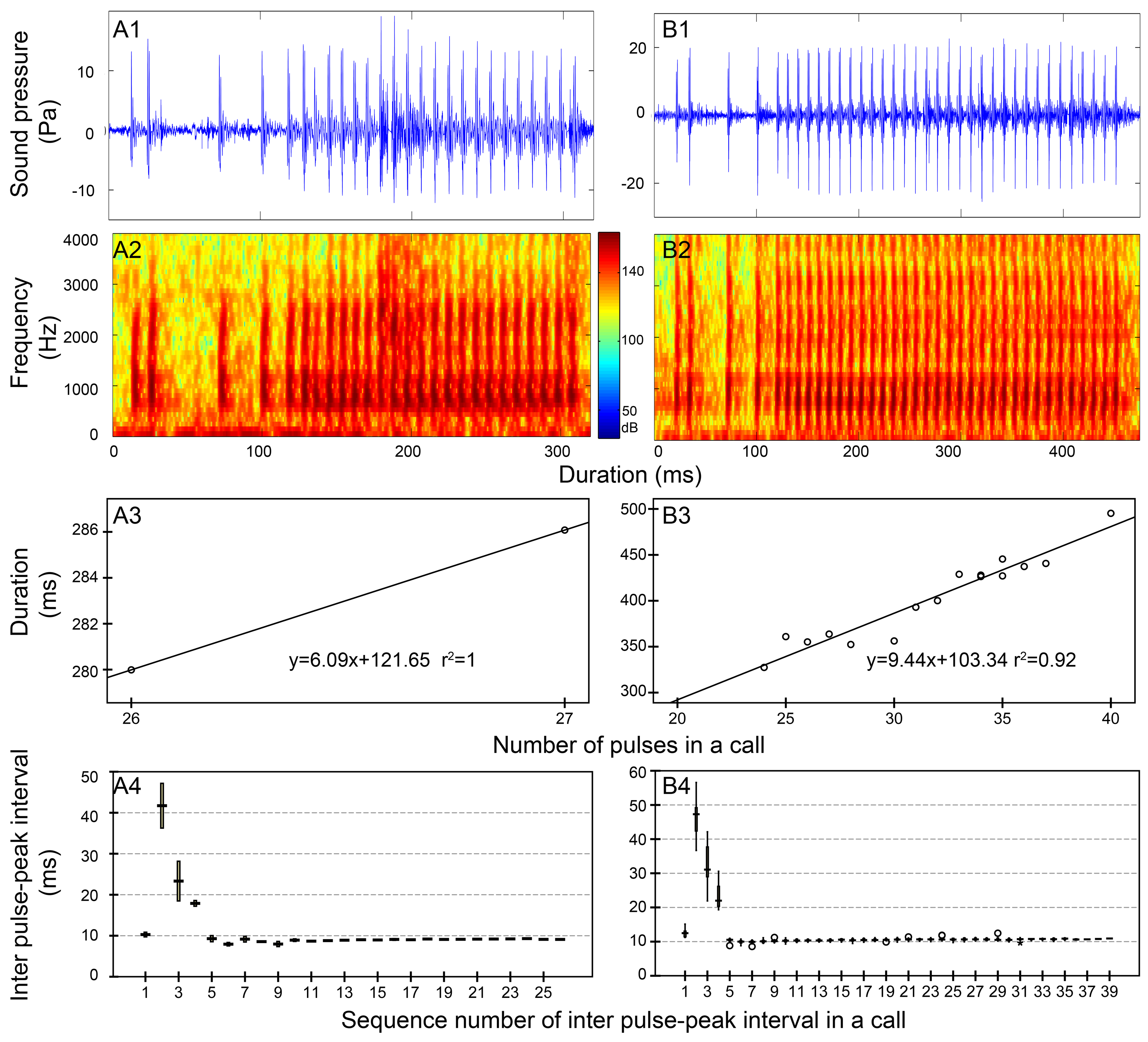

Supplement: Supplemental Information 1 [file peerj-05-3924-s001.zip › Supplemental figures/supplemental figures/Fig.S15.png]

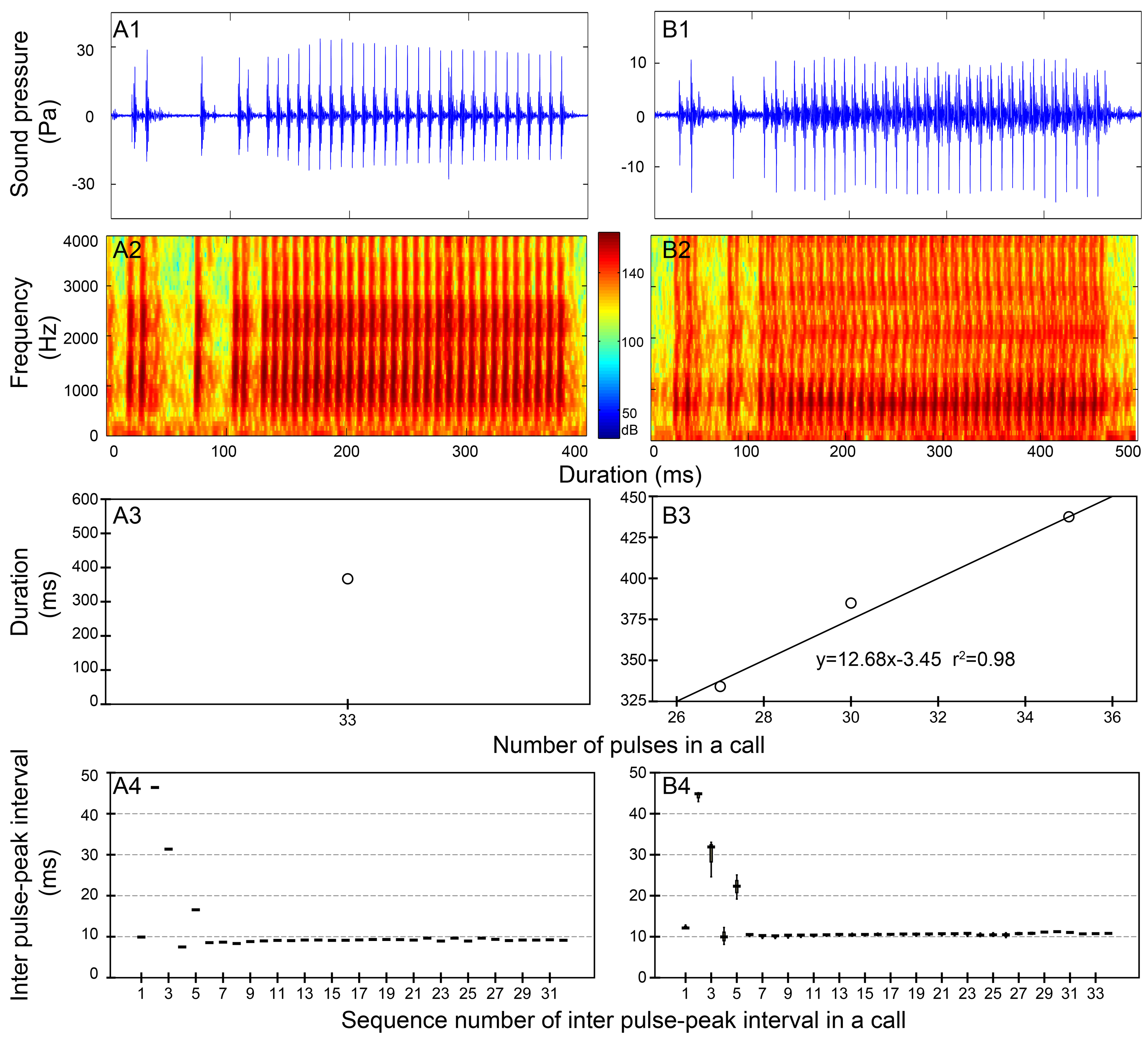

Supplement: Supplemental Information 1 [file peerj-05-3924-s001.zip › Supplemental figures/supplemental figures/Fig.S16.png]

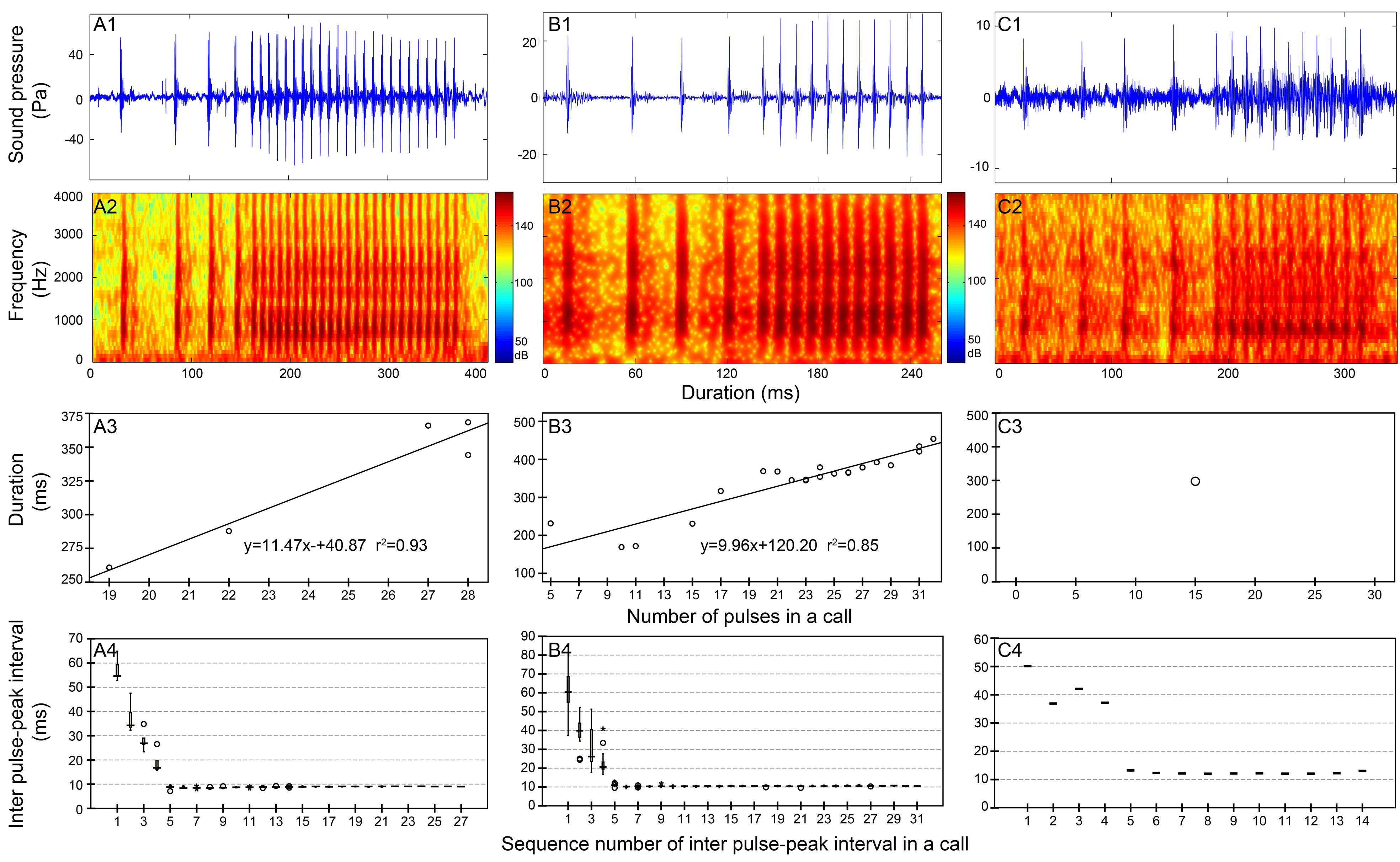

Supplement: Supplemental Information 1 [file peerj-05-3924-s001.zip › Supplemental figures/supplemental figures/Fig.S17.png]

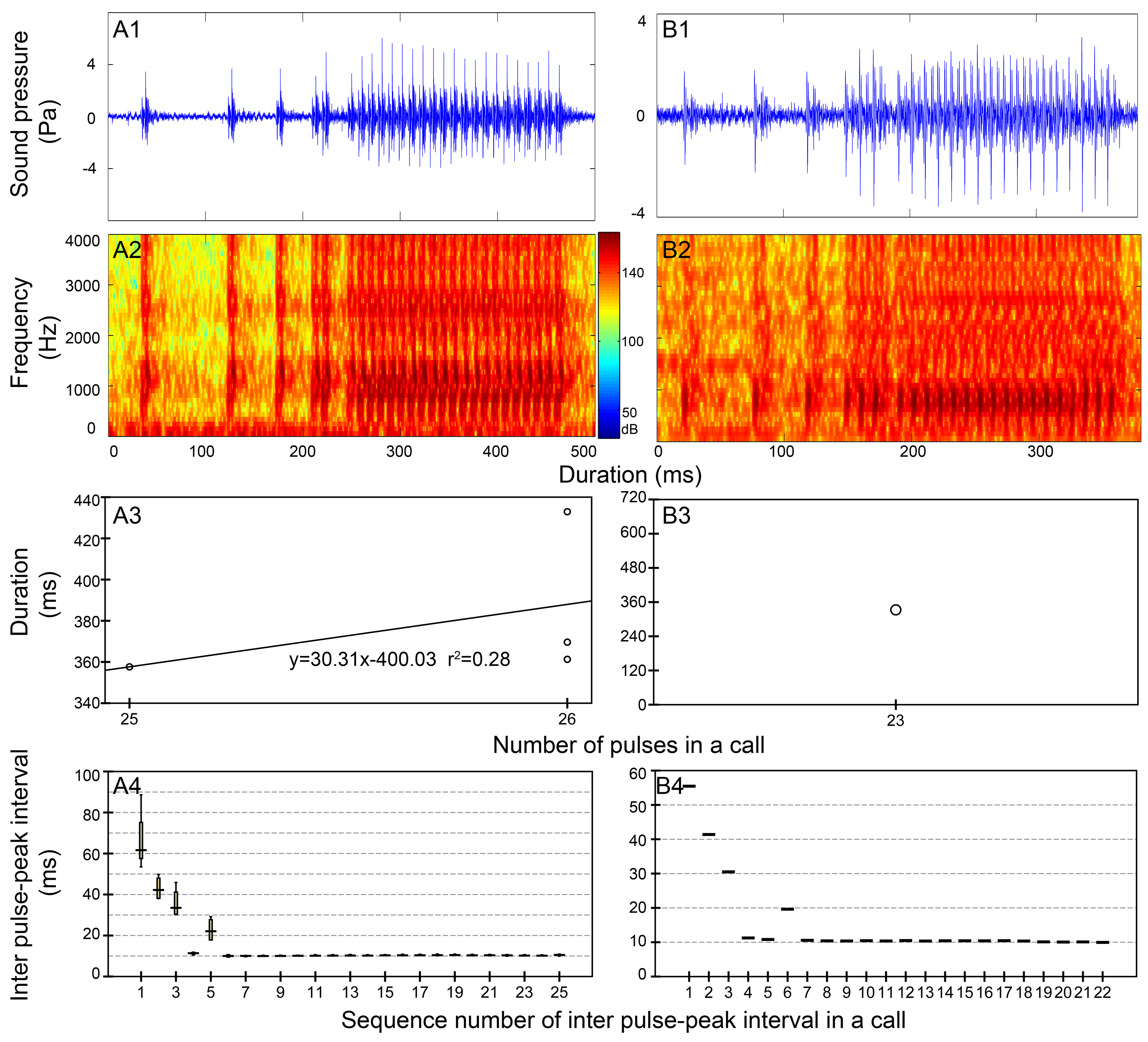

Supplement: Supplemental Information 1 [file peerj-05-3924-s001.zip › Supplemental figures/supplemental figures/Fig.S18.png]

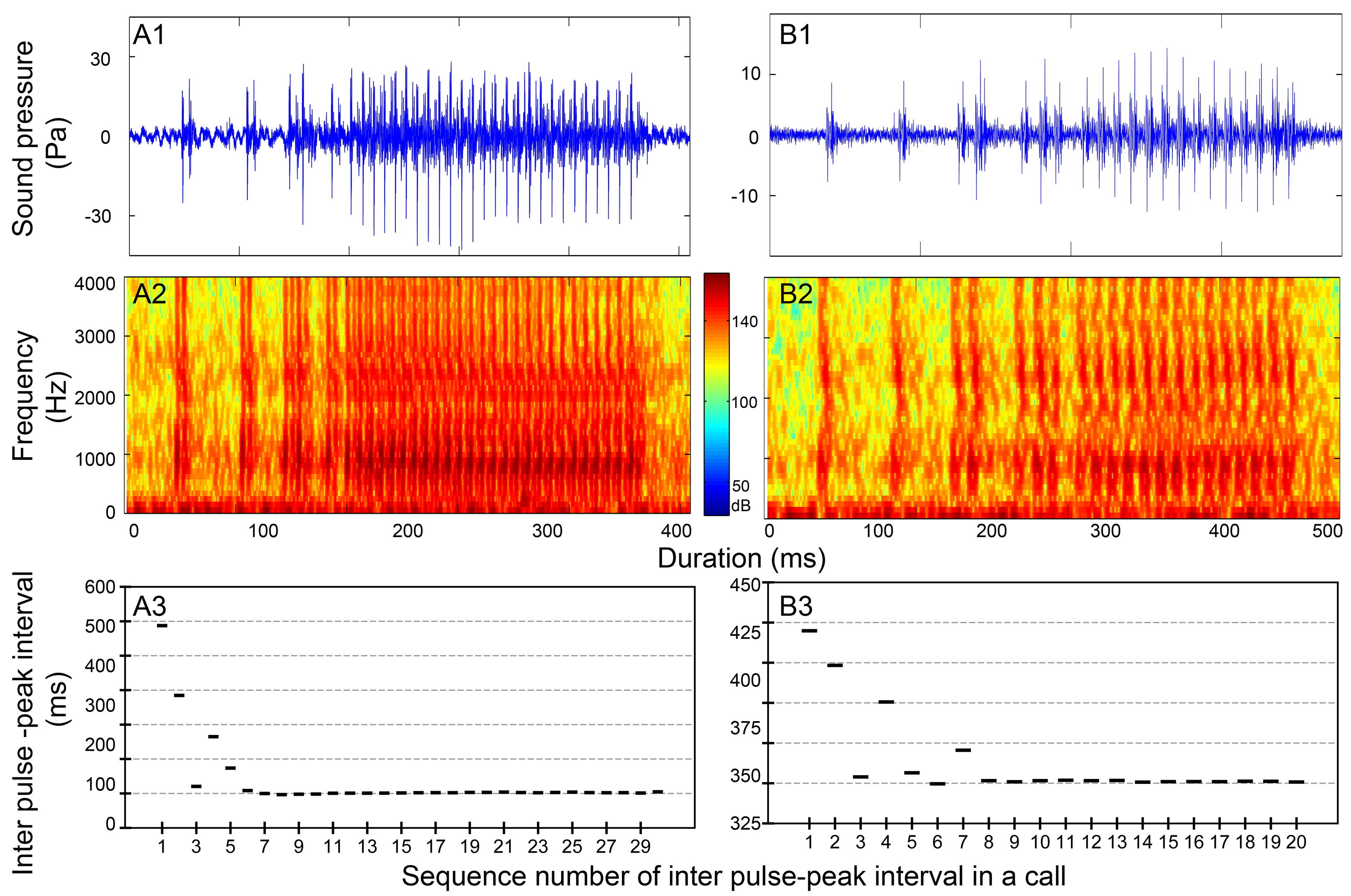

Supplement: Supplemental Information 1 [file peerj-05-3924-s001.zip › Supplemental figures/supplemental figures/Fig.S19.png]

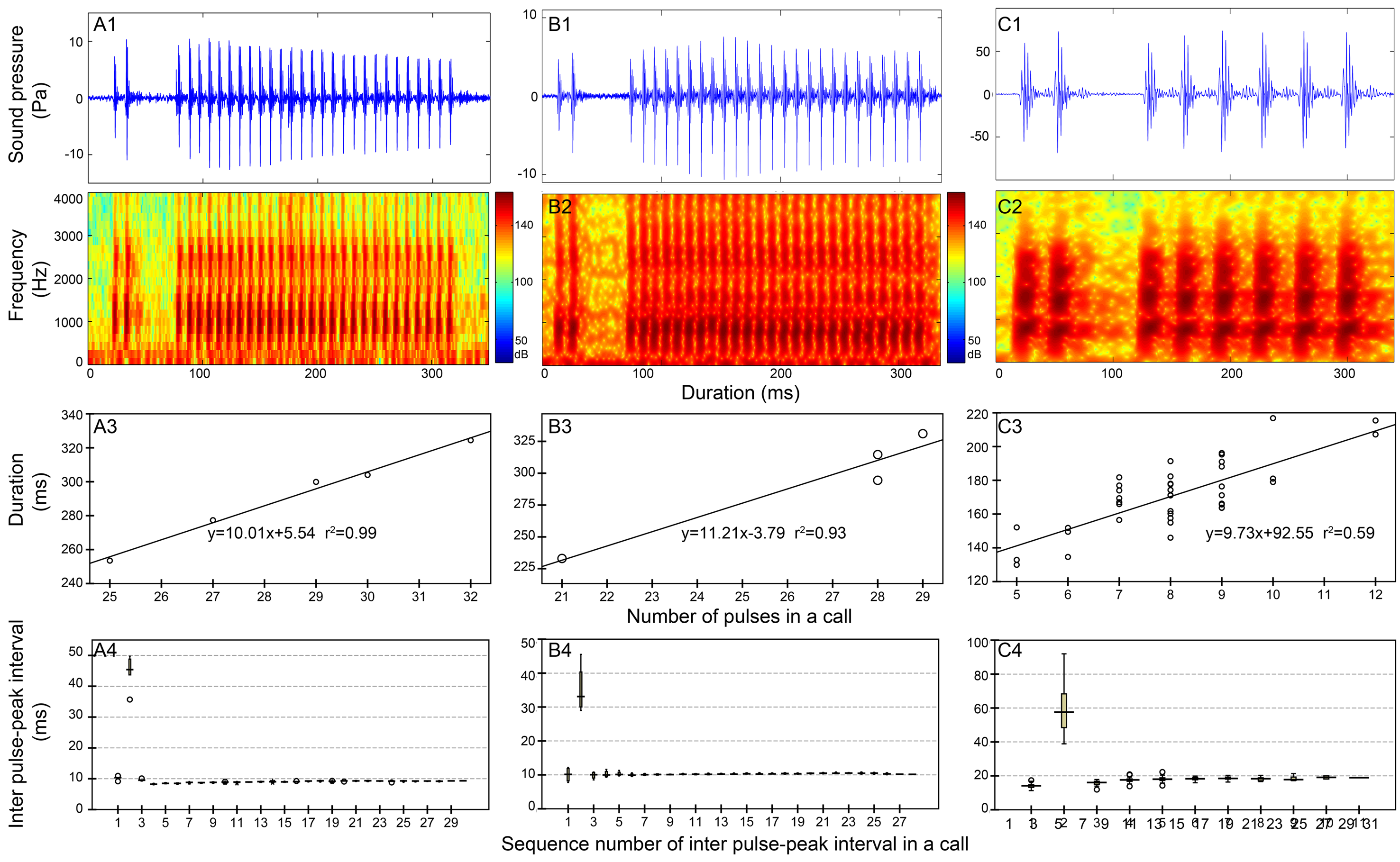

Supplement: Supplemental Information 1 [file peerj-05-3924-s001.zip › Supplemental figures/supplemental figures/Fig.S2.png]

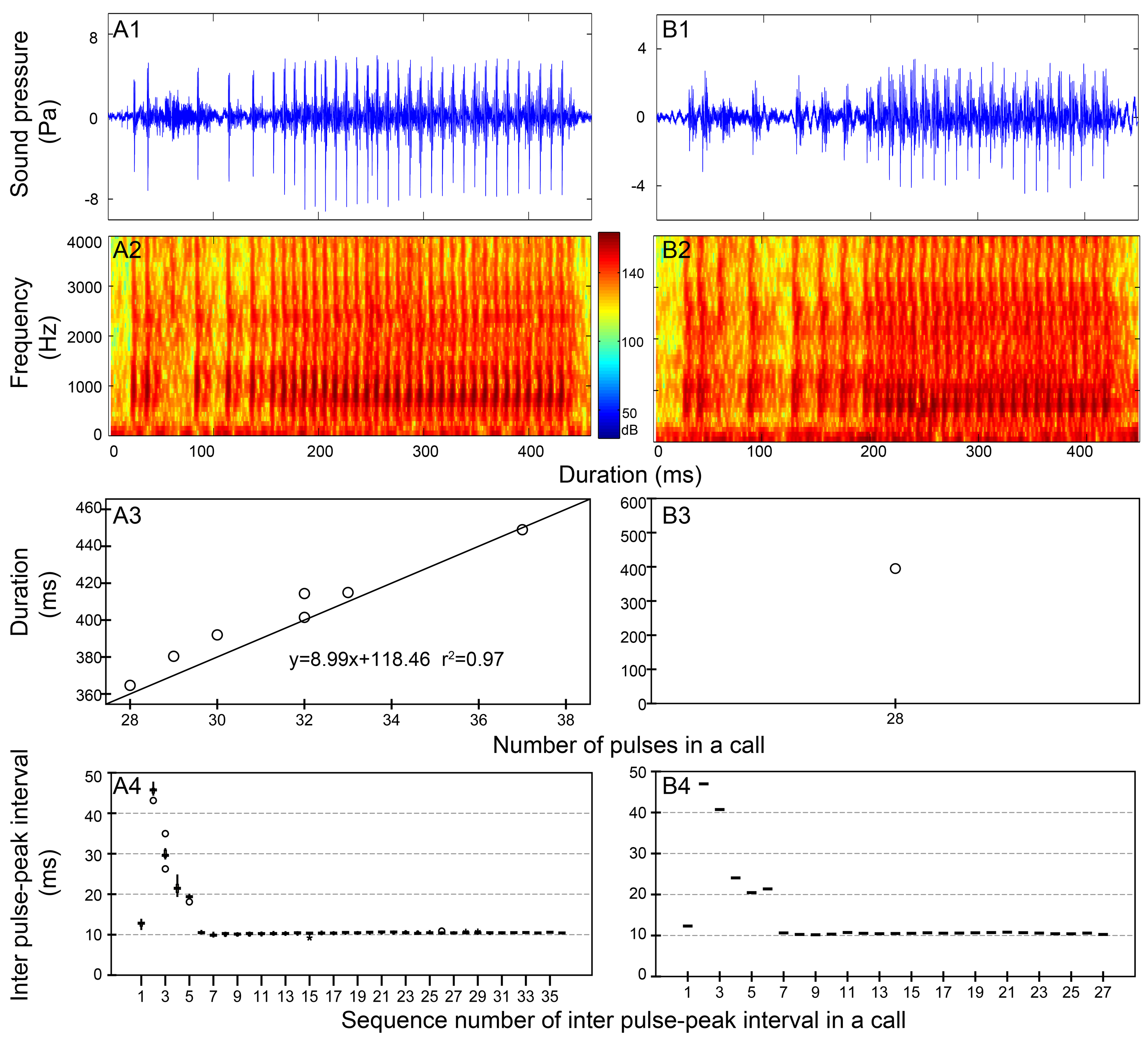

Supplement: Supplemental Information 1 [file peerj-05-3924-s001.zip › Supplemental figures/supplemental figures/Fig.S20.png]

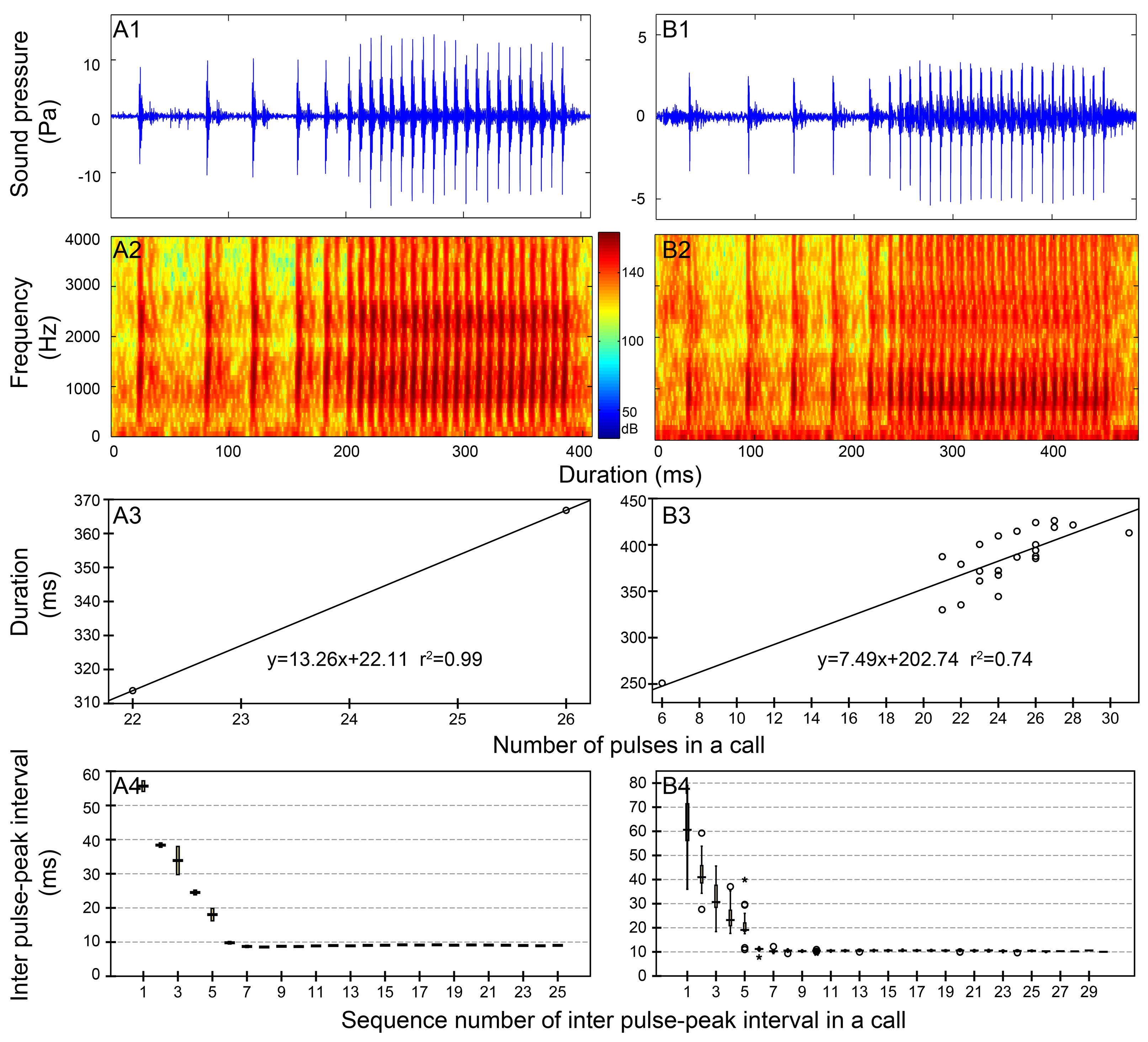

Supplement: Supplemental Information 1 [file peerj-05-3924-s001.zip › Supplemental figures/supplemental figures/Fig.S21.png]

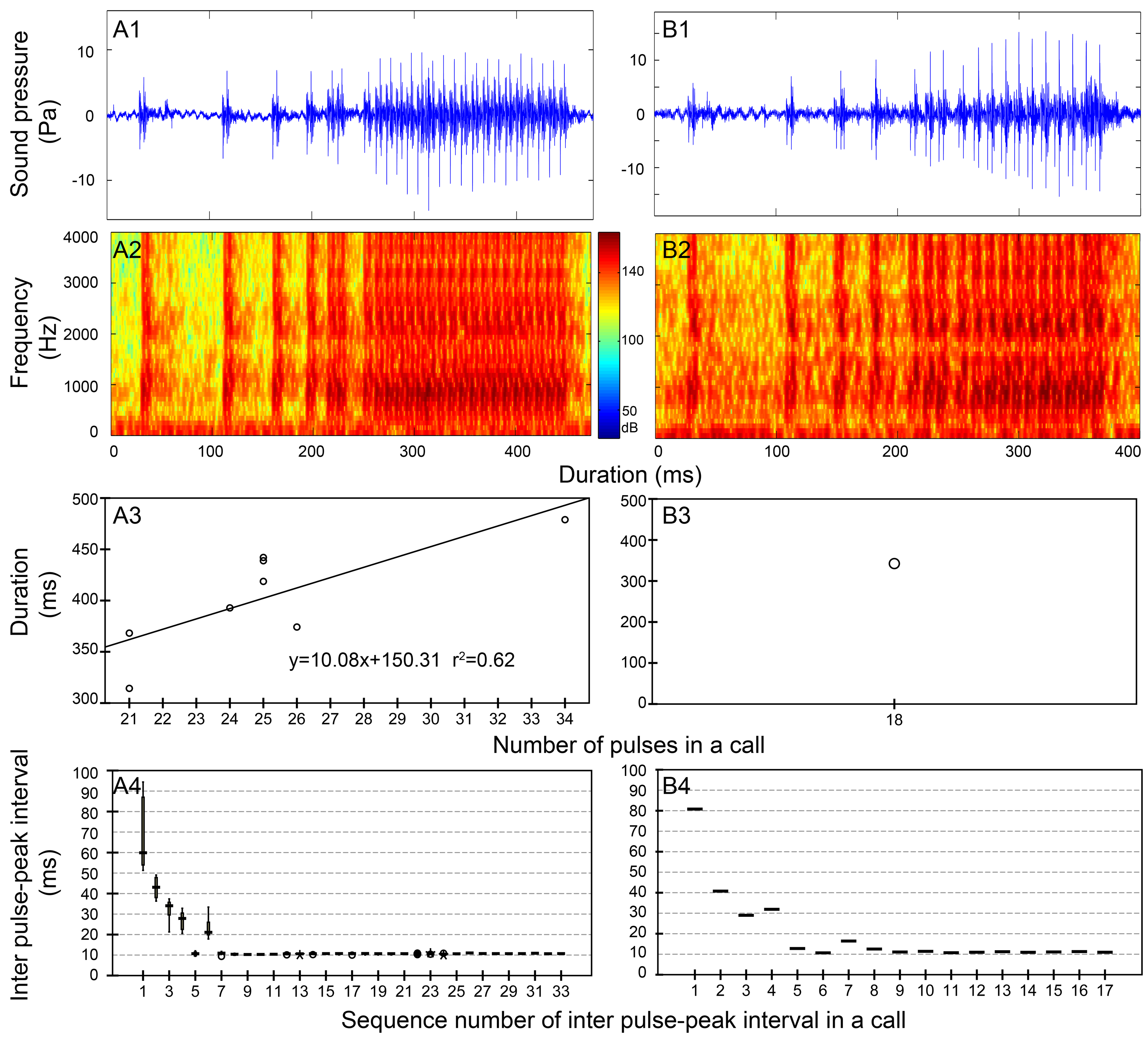

Supplement: Supplemental Information 1 [file peerj-05-3924-s001.zip › Supplemental figures/supplemental figures/Fig.S22.png]

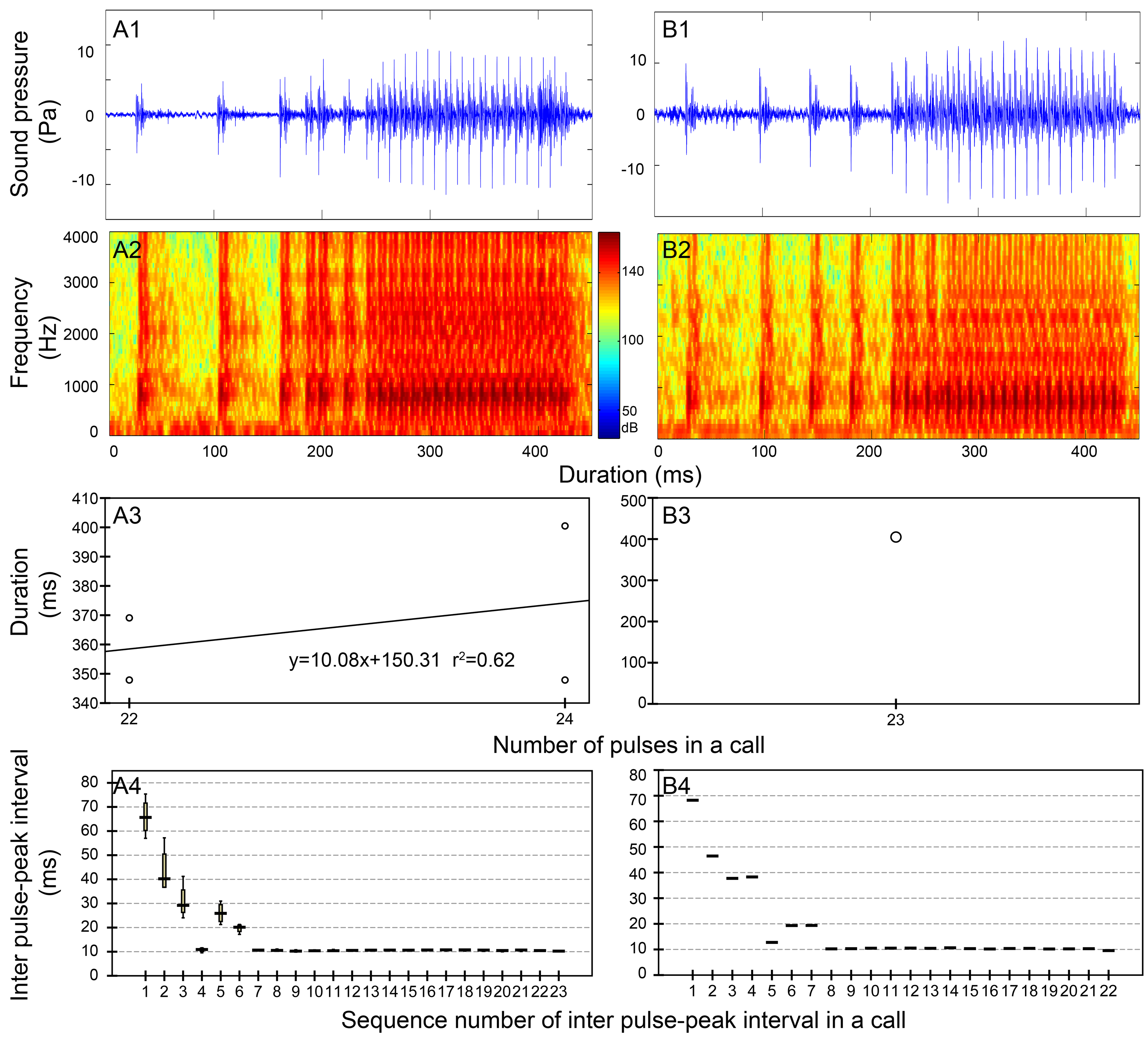

Supplement: Supplemental Information 1 [file peerj-05-3924-s001.zip › Supplemental figures/supplemental figures/Fig.S23.png]

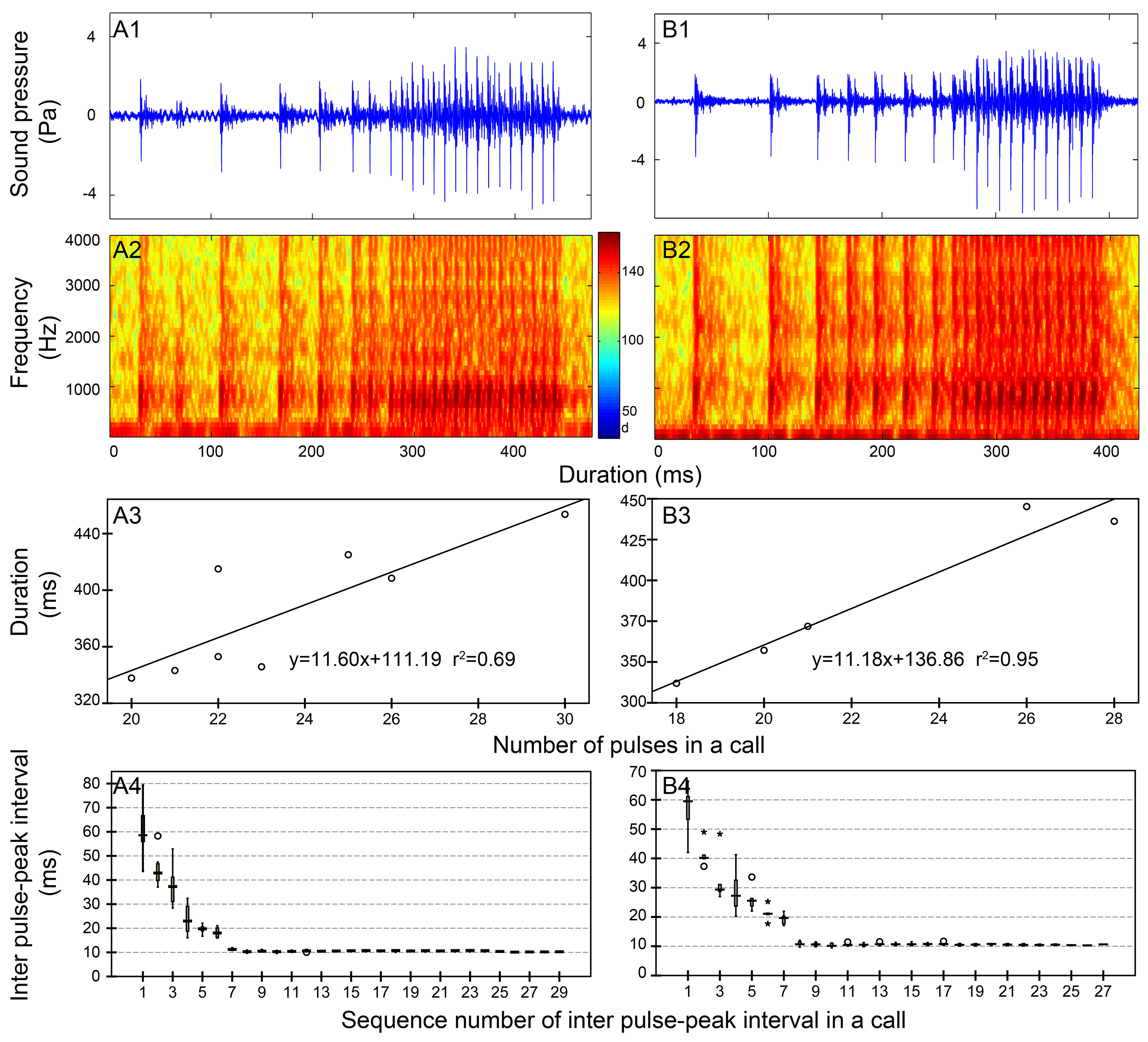

Supplement: Supplemental Information 1 [file peerj-05-3924-s001.zip › Supplemental figures/supplemental figures/Fig.S24.png]

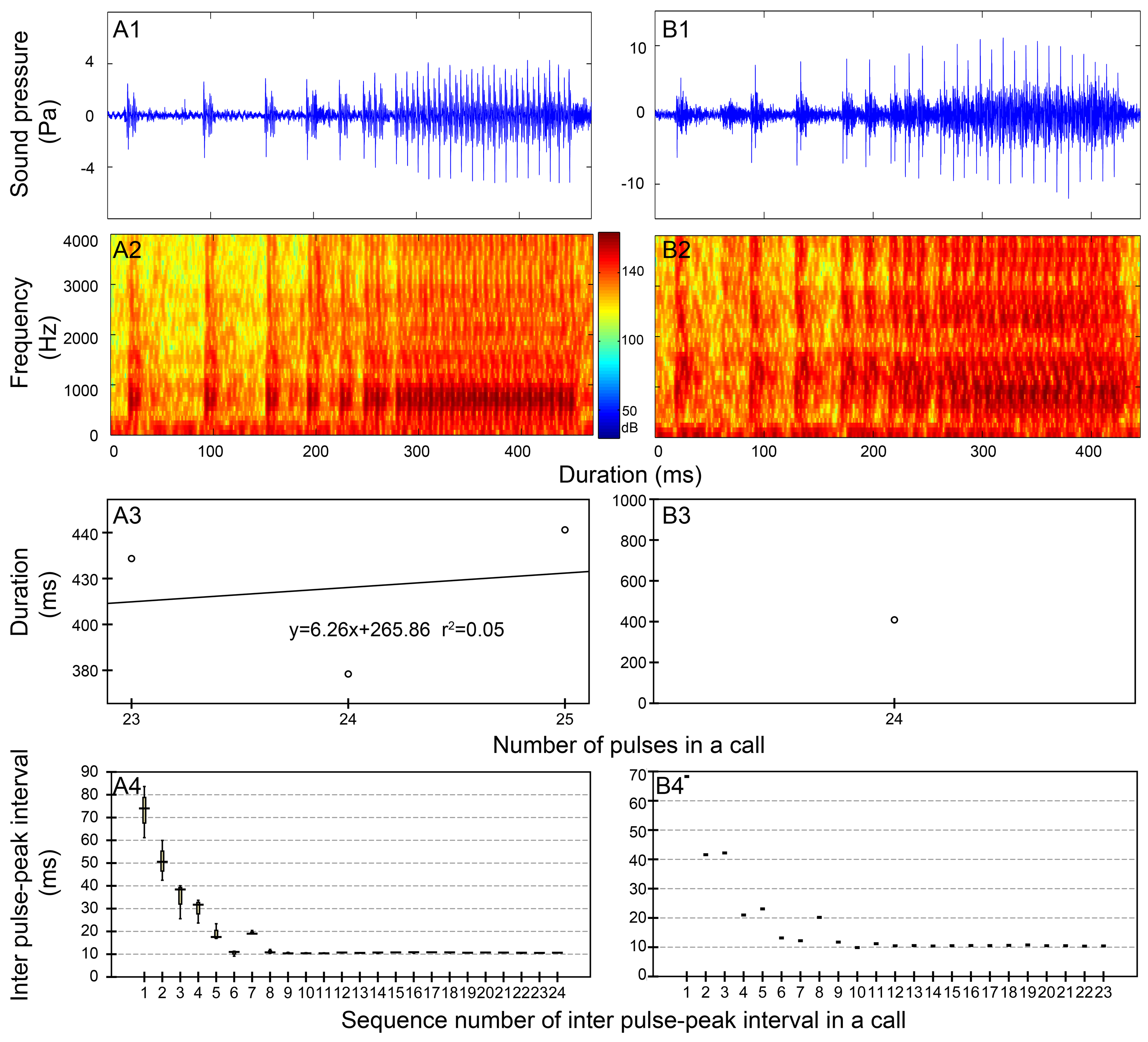

Supplement: Supplemental Information 1 [file peerj-05-3924-s001.zip › Supplemental figures/supplemental figures/Fig.S25.png]

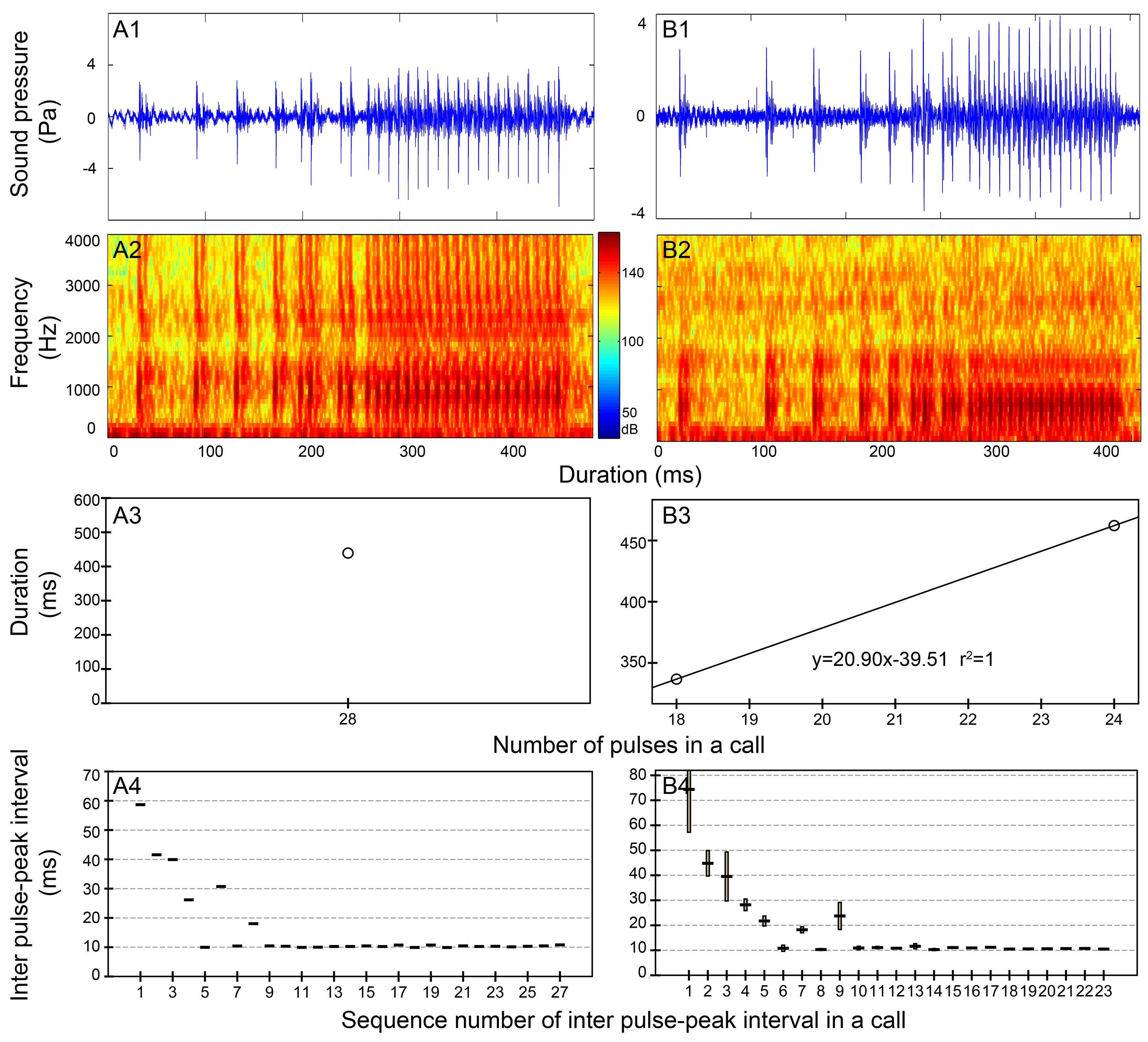

Supplement: Supplemental Information 1 [file peerj-05-3924-s001.zip › Supplemental figures/supplemental figures/Fig.S26.png]

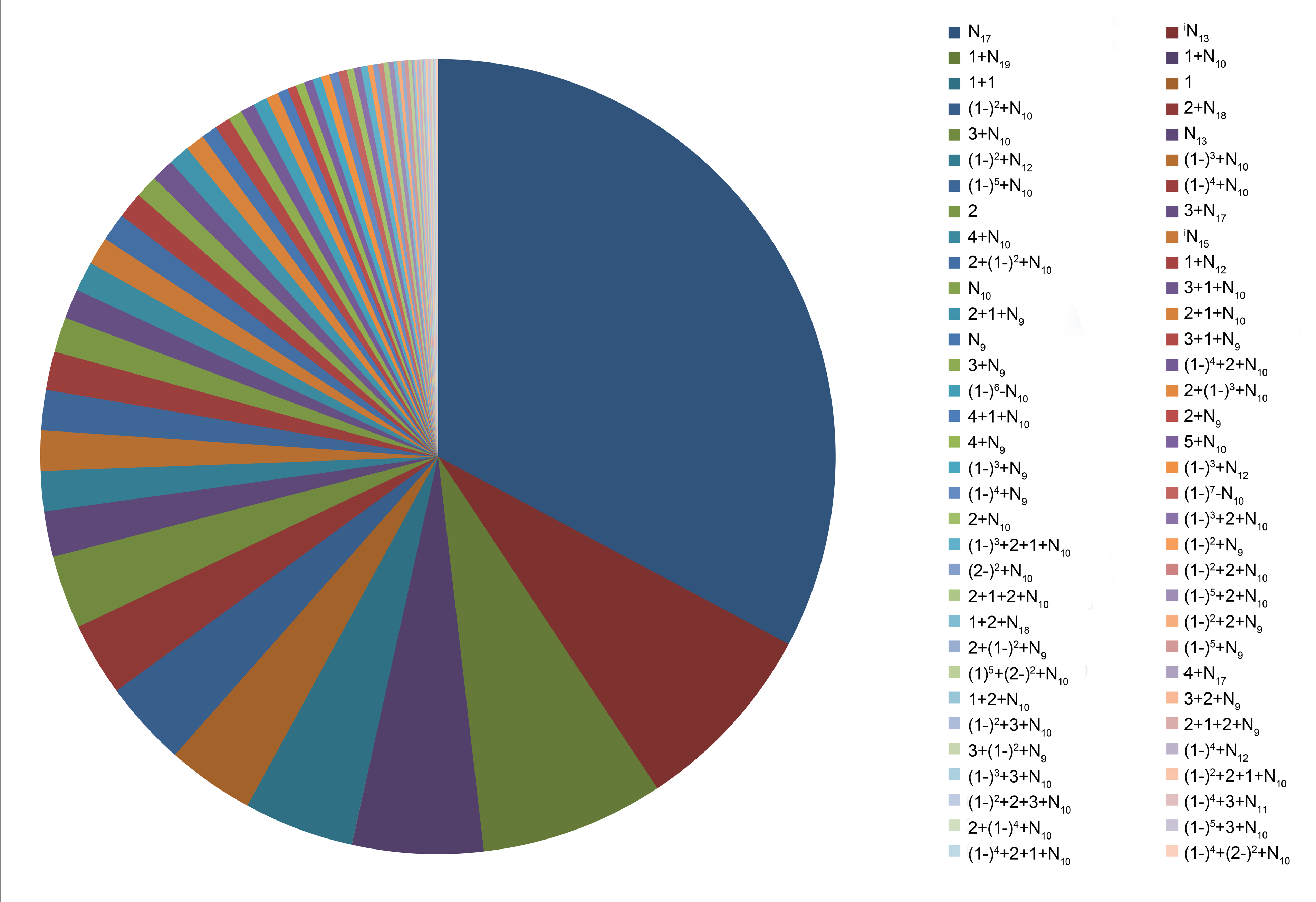

Supplement: Supplemental Information 1 [file peerj-05-3924-s001.zip › Supplemental figures/supplemental figures/Fig.S27.png]

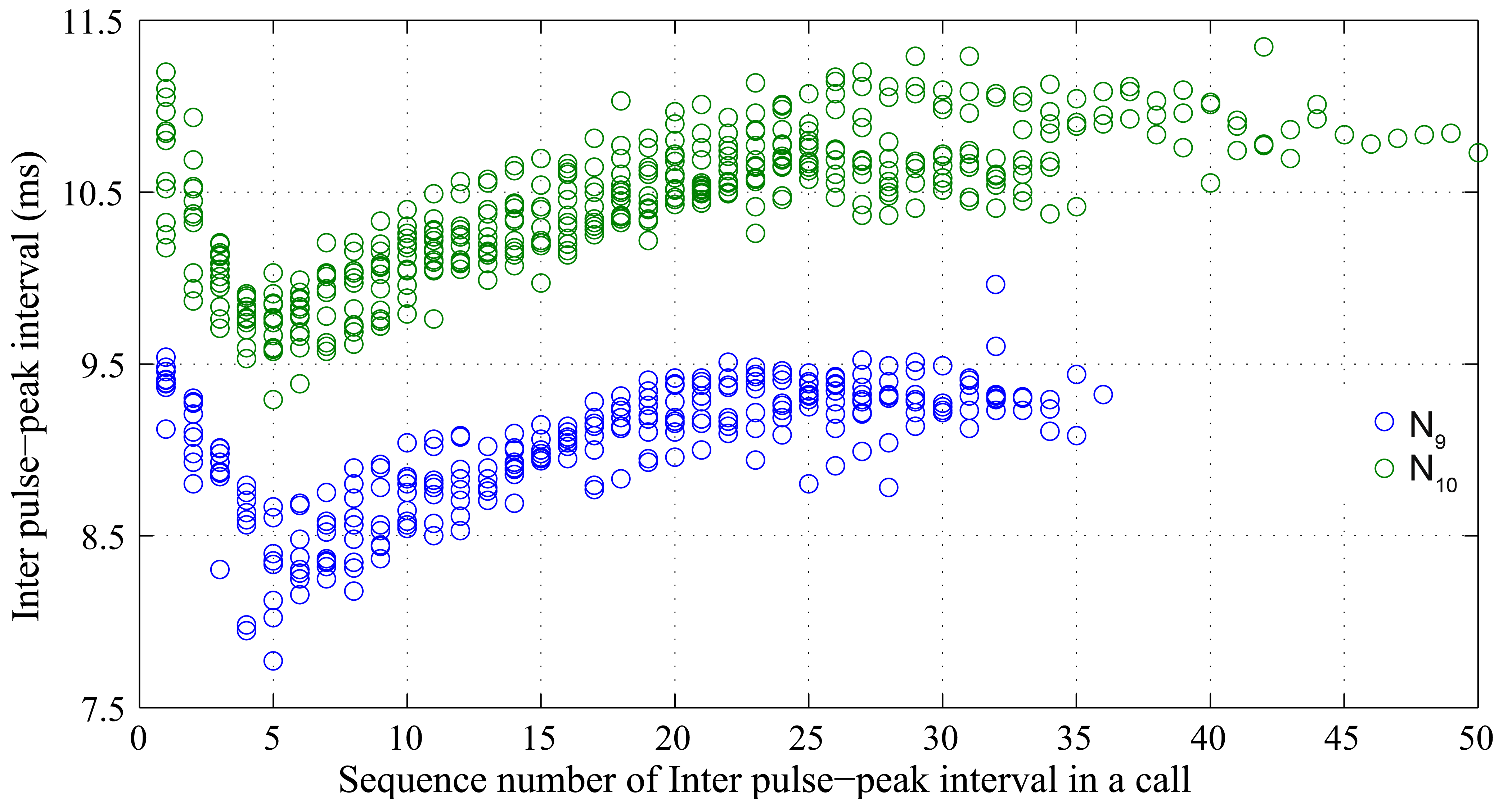

Supplement: Supplemental Information 1 [file peerj-05-3924-s001.zip › Supplemental figures/supplemental figures/Fig.S28.png]

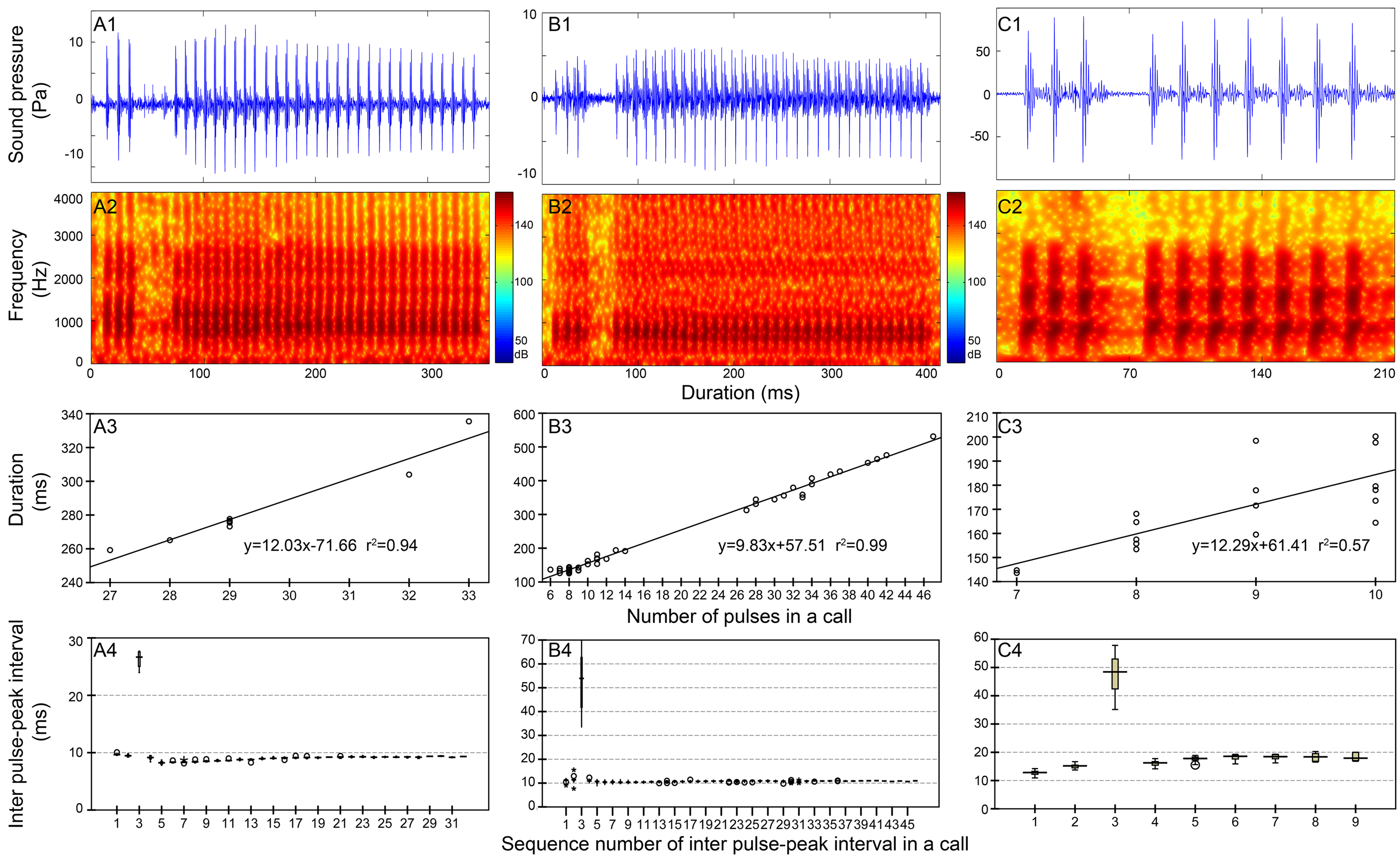

Supplement: Supplemental Information 1 [file peerj-05-3924-s001.zip › Supplemental figures/supplemental figures/Fig.S3.png]

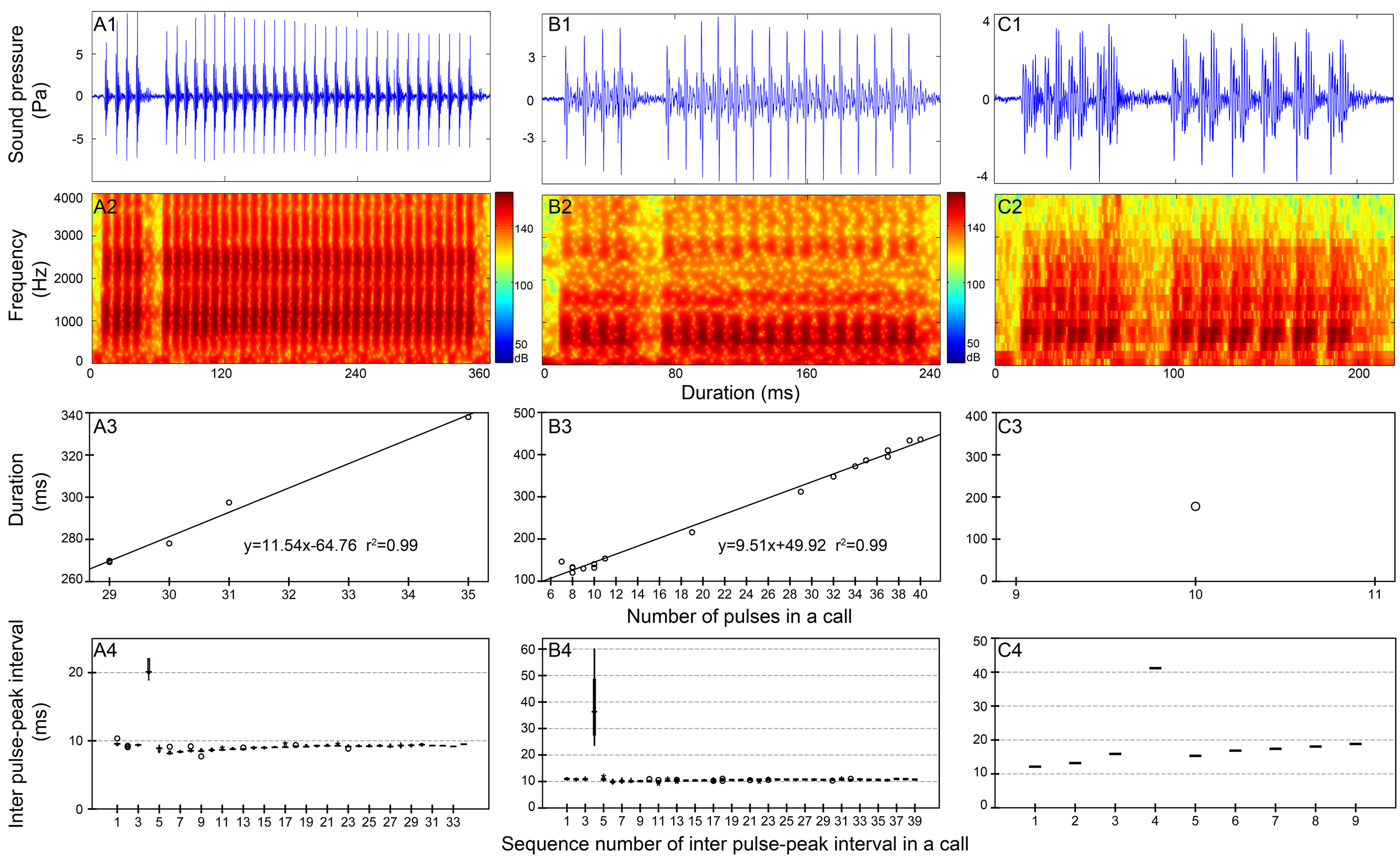

Supplement: Supplemental Information 1 [file peerj-05-3924-s001.zip › Supplemental figures/supplemental figures/Fig.S4.png]

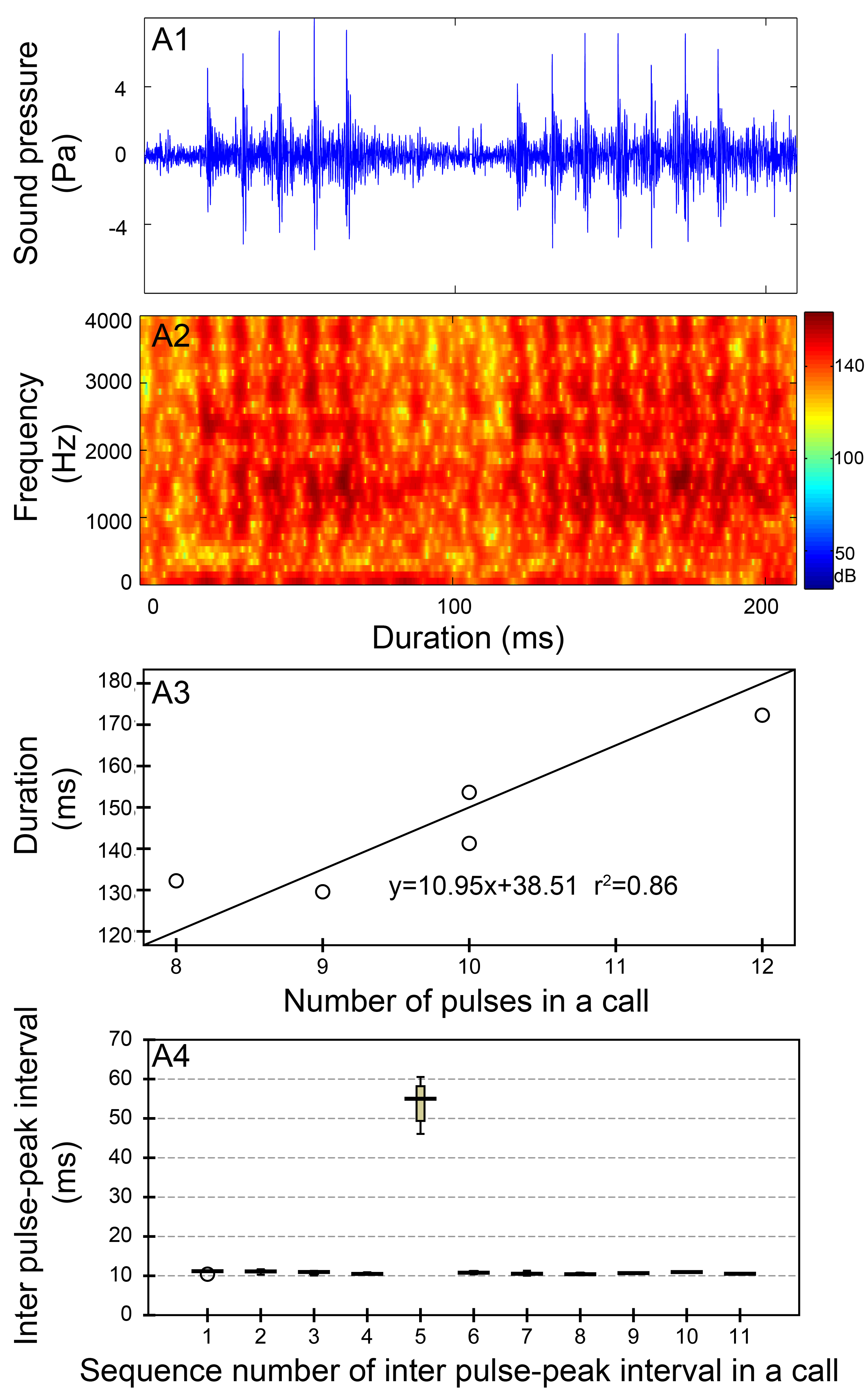

Supplement: Supplemental Information 1 [file peerj-05-3924-s001.zip › Supplemental figures/supplemental figures/Fig.S5.png]

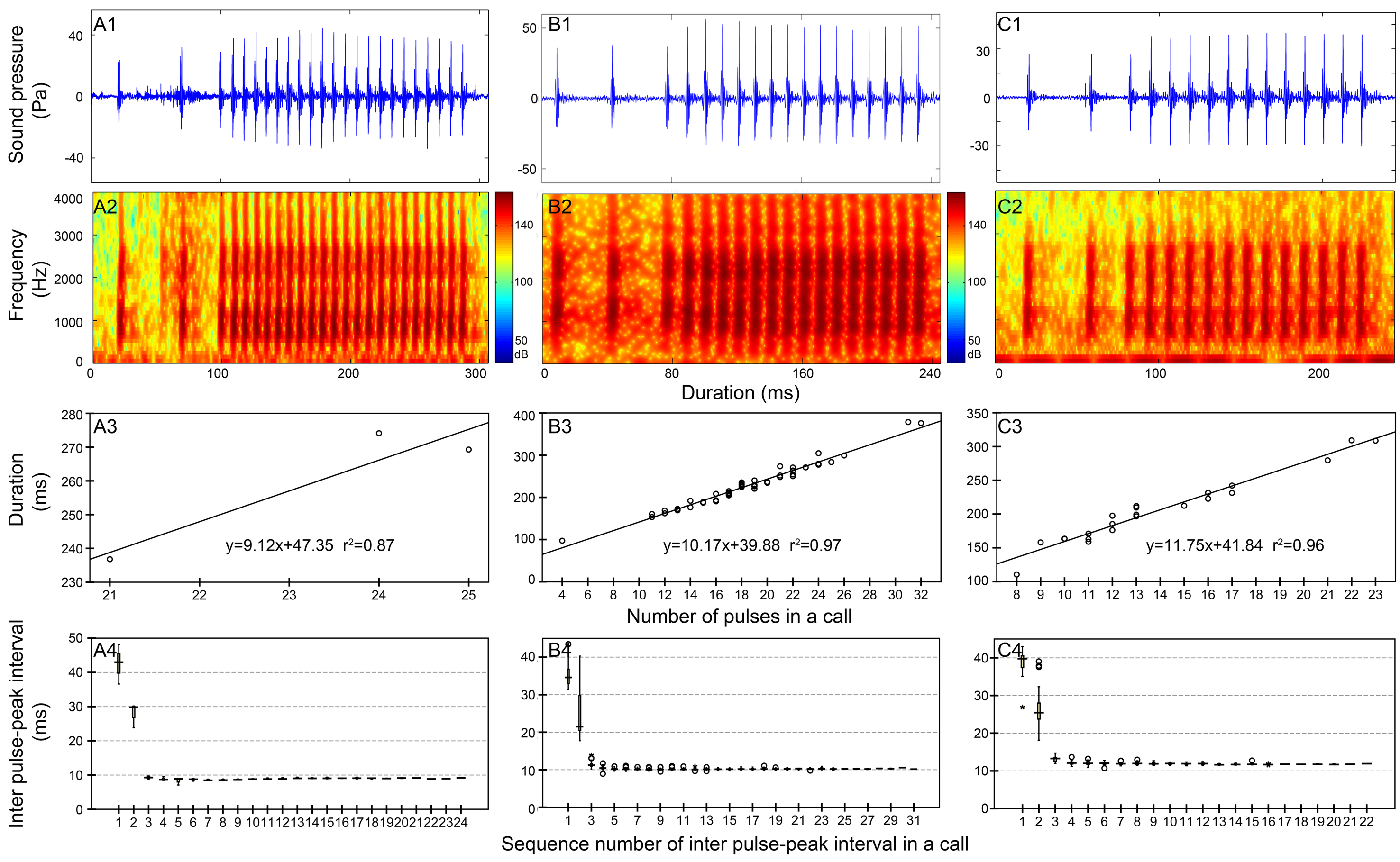

Supplement: Supplemental Information 1 [file peerj-05-3924-s001.zip › Supplemental figures/supplemental figures/Fig.S6.png]

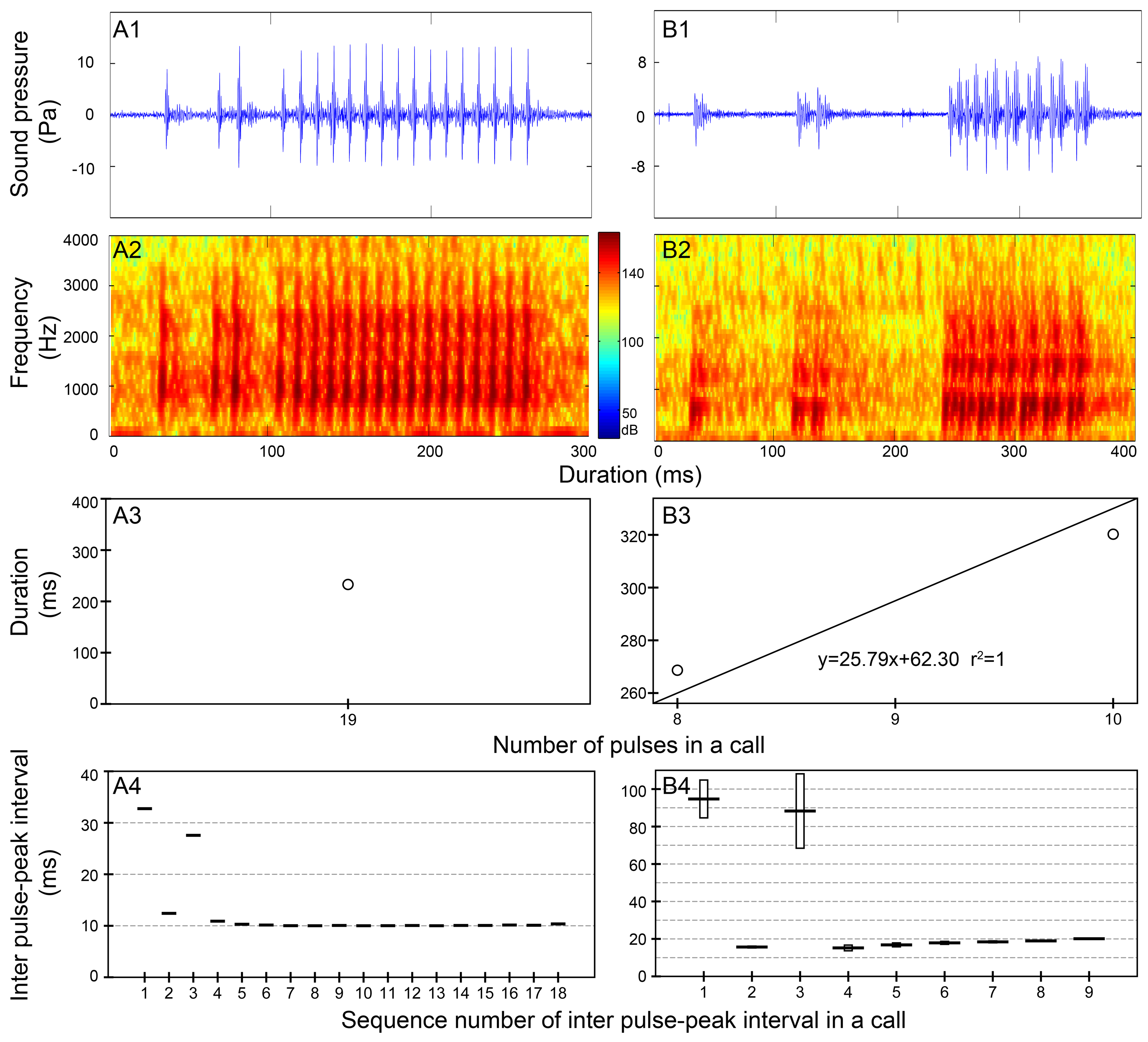

Supplement: Supplemental Information 1 [file peerj-05-3924-s001.zip › Supplemental figures/supplemental figures/Fig.S7.png]

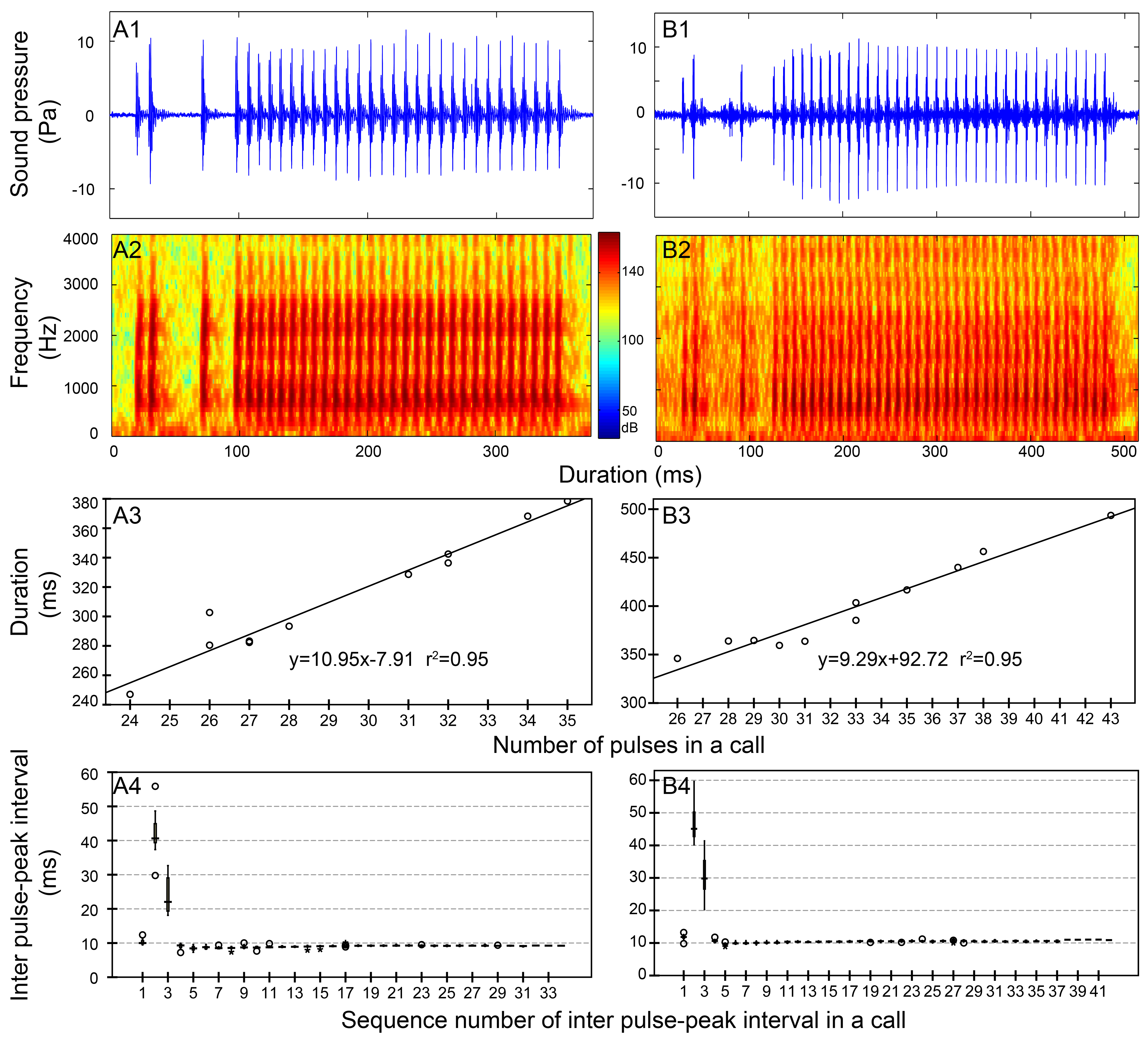

Supplement: Supplemental Information 1 [file peerj-05-3924-s001.zip › Supplemental figures/supplemental figures/Fig.S8.png]

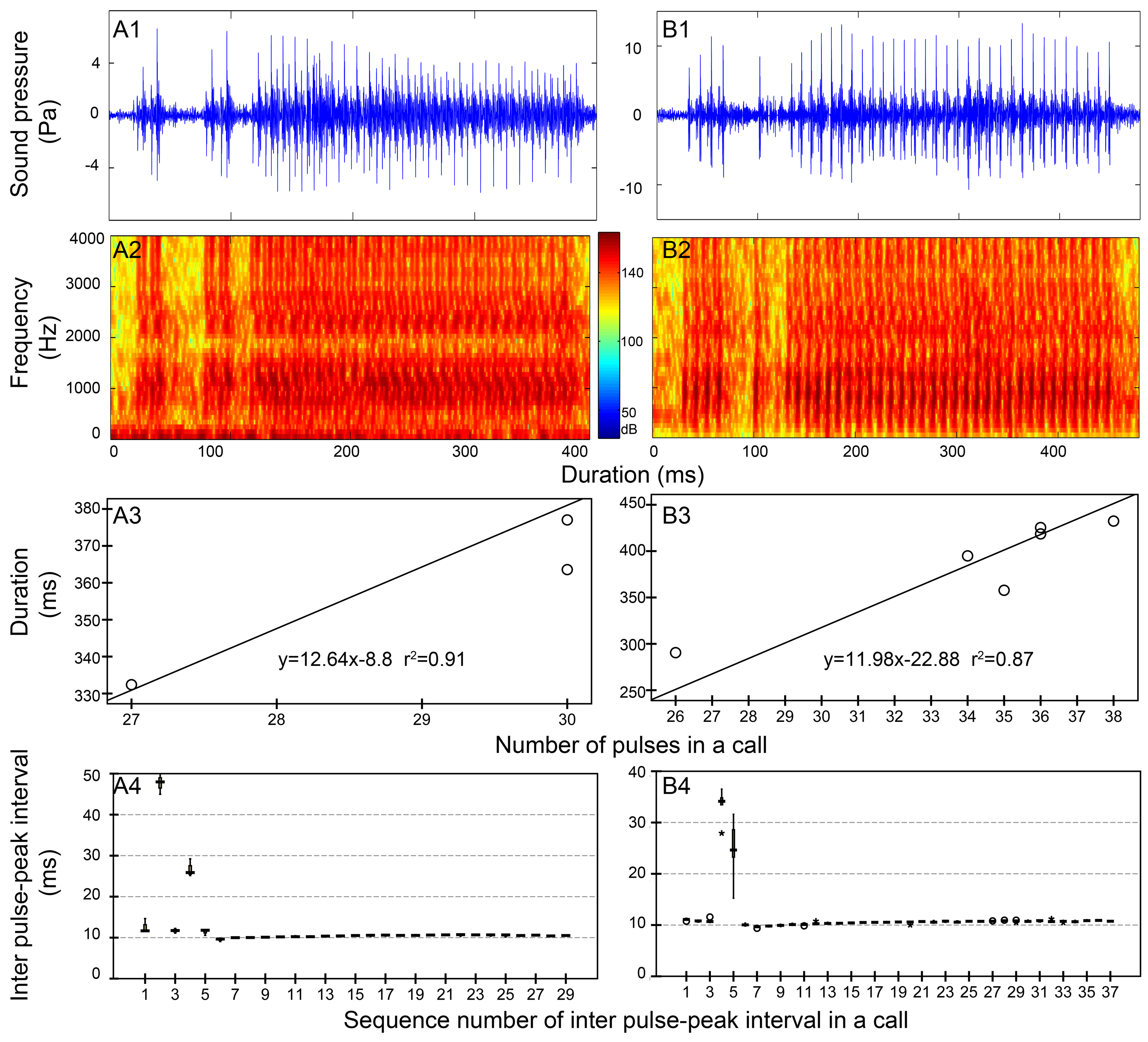

Supplement: Supplemental Information 1 [file peerj-05-3924-s001.zip › Supplemental figures/supplemental figures/Fig.S9.png]
