# Supplementary material for: Diversity of fish sound types in the Pearl River Estuary, China
Source: PeerJ. 2017 Oct 24;5:e3924. doi: 10.7717/peerj.3924 (PMC5659214; doi:10.7717/peerj.3924)
Supplement: Supplemental Information 2 [file peerj-05-3924-s002.zip › Supplemental tables/Supplemental tables/Table S1.docx]

|  |  | Dur | IPPI | τ_95%_ | τ_-3dB_ | τ_-10dB_ | f_p_ | f_c_ | BW_rms_ | Q | SPL_zp_ | SPL_rms_ | EFD | N1 | N2 | N3 |
| --- | --- | --- | --- | --- | --- | --- | --- | --- | --- | --- | --- | --- | --- | --- | --- | --- |
| 1 | P50 | 8.00 | 0.00 | 4.38 | 0.52 | 0.54 | 821 | 1115 | 495 | 2.37 | 153.88 | 145.97 | 172.23 | 50 | 50 | 50 |
|  | QD | 0.00 | 0.00 | 0.59 | 0.04 | 0.04 | 60 | 94 | 62 | 0.21 | 5.20 | 5.45 | 5.85 |  |  |  |
|  | P5 | 8.00 | 0.00 | 3.08 | 0.41 | 0.42 | 718 | 921 | 373 | 1.42 | 141.90 | 133.00 | 159.50 |  |  |  |
|  | P95 | 8.00 | 0.00 | 5.81 | 0.75 | 1.04 | 942 | 1378 | 782 | 2.76 | 162.10 | 153.41 | 179.84 |  |  |  |
| 2 | P50 | 22.31 | 13.41 | 4.06 | 0.52 | 0.90 | 910 | 1219 | 698 | 2.44 | 155.98 | 145.17 | 170.89 | 20 | 20 | 40 |
|  | QD | 0.30 | 0.37 | 0.28 | 0.14 | 0.18 | 219 | 428 | 117 | 0.34 | 4.19 | 2.31 | 2.24 |  |  |  |
|  | P5 | 21.26 | 13.29 | 2.76 | 0.36 | 0.50 | 611 | 728 | 360 | 1.25 | 143.25 | 135.09 | 162.23 |  |  |  |
|  | P95 | 24.69 | 14.97 | 5.93 | 0.86 | 0.98 | 1321 | 1887 | 1035 | 2.64 | 158.81 | 148.99 | 174.52 |  |  |  |
| 1+1 | P50 | 49.87 | 40.87 | 3.44 | 0.42 | 1.33 | 1168 | 1443 | 644 | 2.20 | 148.01 | 138.01 | 163.27 | 63 | 63 | 126 |
|  | QD | 2.58 | 2.68 | 0.67 | 0.04 | 0.33 | 140 | 101 | 87 | 0.34 | 6.33 | 5.27 | 4.76 |  |  |  |
|  | P5 | 41.33 | 32.46 | 2.36 | 0.30 | 0.38 | 787 | 951 | 503 | 1.28 | 127.57 | 120.34 | 147.65 |  |  |  |
|  | P95 | 56.62 | 48.61 | 6.33 | 0.76 | 1.95 | 1292 | 1650 | 1198 | 2.68 | 156.66 | 146.41 | 171.79 |  |  |  |
