# Supplementary material for: Diversity of fish sound types in the Pearl River Estuary, China
Source: PeerJ. 2017 Oct 24;5:e3924. doi: 10.7717/peerj.3924 (PMC5659214; doi:10.7717/peerj.3924)
Supplement: Supplemental Information 2 [file peerj-05-3924-s002.zip › Supplemental tables/Supplemental tables/Table S10.docx]

|  |  | Dur | IPPI | τ_95%_ | τ_-3dB_ | τ_-10dB_ | f_p_ | f_c_ | BW_rms_ | Q | SPL_zp_ | SPL_rms_ | EFD | N1 | N2 | N3 |
| --- | --- | --- | --- | --- | --- | --- | --- | --- | --- | --- | --- | --- | --- | --- | --- | --- |
| 3+1+N_9_ | P50 | 326.5 | 9.15 | 3.19 | 0.35 | 0.35 | 895.5 | 1500.9 | 1460.05 | 0.98 | 129.35 | 121.08 | 145.75 | 9 | 277 | 286 |
|  | QD | 38.89 | 0.22 | 0.37 | 0.09 | 0.1 | 97.3 | 136.1 | 697.89 | 0.37 | 4.36 | 4.45 | 4.13 |  |  |  |
|  | P5 | 266.86 | 8.3 | 2.47 | 0.06 | 0.09 | 800.7 | 1234.2 | 839.76 | 0.47 | 120.88 | 112.93 | 138.26 |  |  |  |
|  | P95 | 380.57 | 17.69 | 4.28 | 0.47 | 0.5 | 1223.3 | 2298.2 | 4705 | 1.83 | 136.85 | 128.4 | 152.83 |  |  |  |
| 3+1+N_10_ | P50 | 390.33 | 10.48 | 4.67 | 0.15 | 0.60 | 866.0 | 1551.6 | 1528.30 | 1.13 | 133.03 | 123.09 | 149.33 | 13 | 440 | 453 |
|  | QD | 27.80 | 0.27 | 1.13 | 0.04 | 0.33 | 68.8 | 301.7 | 341.38 | 0.17 | 5.05 | 3.69 | 4.14 |  |  |  |
|  | P5 | 346.79 | 9.58 | 2.79 | 0.11 | 0.13 | 733.7 | 893.1 | 758.29 | 0.57 | 120.69 | 108.96 | 135.82 |  |  |  |
|  | P95 | 460.82 | 20.33 | 7.48 | 0.46 | 1.60 | 1484.9 | 2458.0 | 4426.83 | 1.47 | 142.59 | 131.38 | 158.20 |  |  |  |
