# Supplementary material for: Diversity of fish sound types in the Pearl River Estuary, China
Source: PeerJ. 2017 Oct 24;5:e3924. doi: 10.7717/peerj.3924 (PMC5659214; doi:10.7717/peerj.3924)
Supplement: Supplemental Information 2 [file peerj-05-3924-s002.zip › Supplemental tables/Supplemental tables/Table S11.docx]

|  |  | Dur | IPPI | τ_95%_ | τ_-3dB_ | τ_-10dB_ | f_p_ | f_c_ | BW_rms_ | Q | SPL_zp_ | SPL_rms_ | EFD | N1 | N2 | N3 |
| --- | --- | --- | --- | --- | --- | --- | --- | --- | --- | --- | --- | --- | --- | --- | --- | --- |
| 3+2+N_9_ | P50 | 308.65 | 9.17 | 4.85 | 0.35 | 0.38 | 755 | 1420 | 963 | 1.42 | 136.01 | 124.92 | 151.50 | 1 | 30 | 31 |
|  | QD | 0.00 | 0.27 | 0.43 | 0.01 | 0.01 | 81 | 83 | 546 | 0.43 | 0.72 | 0.87 | 0.57 |  |  |  |
|  | P5 | 308.65 | 8.11 | 3.53 | 0.32 | 0.35 | 729 | 1340 | 744 | 0.49 | 132.93 | 123.00 | 149.33 |  |  |  |
|  | P95 | 308.65 | 23.67 | 5.50 | 0.43 | 0.44 | 1181 | 2253 | 4279 | 1.92 | 137.54 | 126.87 | 152.71 |  |  |  |
| 3+(1-)^2^+N_9_ | P50 | 303.51 | 9.36 | 4.79 | 0.38 | 0.36 | 862 | 1488 | 2421 | 0.62 | 123.93 | 114.06 | 140.83 | 1 | 29 | 30 |
|  | QD | 0.00 | 0.33 | 0.69 | 0.09 | 0.13 | 66 | 143 | 595 | 0.08 | 0.62 | 0.94 | 0.89 |  |  |  |
|  | P5 | 303.51 | 8.55 | 2.91 | 0.04 | 0.04 | 720 | 1279 | 1675 | 0.47 | 121.71 | 112.24 | 139.76 |  |  |  |
|  | P95 | 303.51 | 20.76 | 7.02 | 0.80 | 1.02 | 1168 | 3544 | 7324 | 0.81 | 127.05 | 117.93 | 142.48 |  |  |  |
