# Supplementary material for: Diversity of fish sound types in the Pearl River Estuary, China
Source: PeerJ. 2017 Oct 24;5:e3924. doi: 10.7717/peerj.3924 (PMC5659214; doi:10.7717/peerj.3924)
Supplement: Supplemental Information 2 [file peerj-05-3924-s002.zip › Supplemental tables/Supplemental tables/Table S12.docx]

|  |  | Dur | IPPI | τ_95%_ | τ_-3dB_ | τ_-10dB_ | f_p_ | f_c_ | BW_rms_ | Q | SPL_zp_ | SPL_rms_ | EFD | N1 | N2 | N3 |
| --- | --- | --- | --- | --- | --- | --- | --- | --- | --- | --- | --- | --- | --- | --- | --- | --- |
| (1-)^3^+N_9_ | P50 | 296.26 | 8.96 | 3.00 | 0.42 | 0.43 | 822 | 1345 | 902 | 1.25 | 135.17 | 128.02 | 152.24 | 5 | 125 | 130 |
|  | QD | 40.37 | 0.22 | 0.49 | 0.10 | 0.11 | 53 | 202 | 431 | 0.39 | 2.93 | 2.50 | 2.15 |  |  |  |
|  | P5 | 272.18 | 8.40 | 2.11 | 0.14 | 0.15 | 773 | 868 | 637 | 0.54 | 127.07 | 118.09 | 143.80 |  |  |  |
|  | P95 | 361.45 | 38.91 | 4.17 | 0.99 | 1.26 | 1217 | 2082 | 3808 | 2.06 | 138.10 | 130.16 | 153.70 |  |  |  |
| (1-)^3^+N_10_ | P50 | 222.79 | 10.53 | 3.32 | 0.34 | 0.42 | 877 | 1312 | 1089 | 1.12 | 137.37 | 127.48 | 154.07 | 23 | 423 | 446 |
|  | QD | 90.68 | 0.36 | 0.71 | 0.15 | 0.35 | 111 | 190 | 486 | 0.41 | 7.32 | 8.50 | 8.03 |  |  |  |
|  | P5 | 94.16 | 9.81 | 2.07 | 0.10 | 0.11 | 729 | 983 | 606 | 0.53 | 121.69 | 110.44 | 135.83 |  |  |  |
|  | P95 | 479.54 | 36.30 | 5.98 | 1.03 | 1.92 | 1350 | 2228 | 3849 | 2.29 | 152.19 | 142.00 | 167.04 |  |  |  |
| (1-)^3^+N_12_ | P50 | 202.81 | 11.91 | 3.51 | 0.46 | 0.74 | 884 | 1222 | 666 | 1.83 | 148.94 | 139.42 | 165.05 | 5 | 61 | 66 |
|  | QD | 14.04 | 3.00 | 0.59 | 0.14 | 0.41 | 29 | 184 | 280 | 0.72 | 13.37 | 11.91 | 11.60 |  |  |  |
|  | P5 | 189.03 | 11.17 | 2.47 | 0.39 | 0.44 | 817 | 910 | 524 | 0.43 | 117.25 | 109.76 | 135.08 |  |  |  |
|  | P95 | 241.36 | 40.51 | 4.67 | 0.93 | 1.59 | 1235 | 1420 | 2877 | 2.70 | 152.15 | 143.02 | 167.60 |  |  |  |
