# Supplementary material for: Diversity of fish sound types in the Pearl River Estuary, China
Source: PeerJ. 2017 Oct 24;5:e3924. doi: 10.7717/peerj.3924 (PMC5659214; doi:10.7717/peerj.3924)
Supplement: Supplemental Information 2 [file peerj-05-3924-s002.zip › Supplemental tables/Supplemental tables/Table S13.docx]

|  |  | Dur | IPPI | τ_95%_ | τ_-3dB_ | τ_-10dB_ | f_p_ | f_c_ | BW_rms_ | Q | SPL_zp_ | SPL_rms_ | EFD | N1 | N2 | N3 |
| --- | --- | --- | --- | --- | --- | --- | --- | --- | --- | --- | --- | --- | --- | --- | --- | --- |
| (1-)^2^+2+N_9_ | P50 | 355.98 | 9.06 | 3.77 | 0.65 | 0.44 | 786 | 1174 | 1758 | 0.65 | 126.19 | 117.99 | 143.68 | 2 | 61 | 63 |
|  | QD | 11.27 | 0.21 | 0.56 | 0.22 | 0.15 | 47 | 151 | 733 | 0.18 | 0.86 | 1.29 | 0.93 |  |  |  |
|  | P5 | 344.71 | 8.24 | 2.88 | 0.08 | 0.16 | 712 | 972 | 1000 | 0.40 | 124.22 | 115.76 | 142.02 |  |  |  |
|  | P95 | 367.24 | 32.71 | 5.62 | 1.34 | 1.35 | 889 | 1755 | 3717 | 1.11 | 127.98 | 120.64 | 145.44 |  |  |  |
| (1-)^2^+2+N_10_ | P50 | 389.80 | 10.58 | 5.32 | 0.23 | 0.20 | 856 | 1048 | 1404 | 0.85 | 127.70 | 118.08 | 145.04 | 3 | 79 | 82 |
|  | QD | 58.70 | 0.21 | 0.97 | 0.15 | 0.05 | 112 | 538 | 608 | 0.13 | 3.34 | 4.66 | 3.80 |  |  |  |
|  | P5 | 360.90 | 10.03 | 3.89 | 0.11 | 0.12 | 693 | 893 | 845 | 0.51 | 121.19 | 108.54 | 136.47 |  |  |  |
|  | P95 | 478.30 | 48.37 | 7.65 | 0.55 | 0.60 | 1561 | 2600 | 3988 | 1.08 | 132.51 | 122.65 | 148.98 |  |  |  |
