# Supplementary material for: Diversity of fish sound types in the Pearl River Estuary, China
Source: PeerJ. 2017 Oct 24;5:e3924. doi: 10.7717/peerj.3924 (PMC5659214; doi:10.7717/peerj.3924)
Supplement: Supplemental Information 2 [file peerj-05-3924-s002.zip › Supplemental tables/Supplemental tables/Table S14.docx]

|  |  | Dur | IPPI | τ_95%_ | τ_-3dB_ | τ_-10dB_ | f_p_ | f_c_ | BW_rms_ | Q | SPL_zp_ | SPL_rms_ | EFD | N1 | N2 | N3 |
| --- | --- | --- | --- | --- | --- | --- | --- | --- | --- | --- | --- | --- | --- | --- | --- | --- |
| (1-)^2^+3+N_10_ | P50 | 287.66 | 10.55 | 7.32 | 0.13 | 0.13 | 884 | 1788 | 1741 | 1.03 | 128.48 | 116.71 | 145.30 | 1 | 22 | 23 |
|  | QD | 0.00 | 0.42 | 0.15 | 0.01 | 0.01 | 27 | 189 | 186 | 0.08 | 1.01 | 0.87 | 1.12 |  |  |  |
|  | P5 | 287.66 | 9.78 | 6.10 | 0.11 | 0.11 | 627 | 1506 | 1451 | 0.51 | 124.89 | 113.00 | 141.31 |  |  |  |
|  | P95 | 287.66 | 33.88 | 7.67 | 0.16 | 0.15 | 919 | 2575 | 4953 | 1.15 | 129.56 | 117.77 | 146.31 |  |  |  |
