# Supplementary material for: Diversity of fish sound types in the Pearl River Estuary, China
Source: PeerJ. 2017 Oct 24;5:e3924. doi: 10.7717/peerj.3924 (PMC5659214; doi:10.7717/peerj.3924)
Supplement: Supplemental Information 2 [file peerj-05-3924-s002.zip › Supplemental tables/Supplemental tables/Table S15.docx]

|  |  | Dur | IPPI | τ_95%_ | τ_-3dB_ | τ_-10dB_ | f_p_ | f_c_ | BW_rms_ | Q | SPL_zp_ | SPL_rms_ | EFD | N1 | N2 | N3 |
| --- | --- | --- | --- | --- | --- | --- | --- | --- | --- | --- | --- | --- | --- | --- | --- | --- |
| 2+(1-)^2^+N_9_ | P50 | 293.04 | 9.09 | 3.60 | 0.31 | 0.25 | 870.0 | 1403.9 | 1727.30 | 0.88 | 127.62 | 117.03 | 141.98 | 2 | 51 | 53 |
|  | QD | 6.96 | 0.23 | 0.20 | 0.10 | 0.10 | 113.0 | 188.6 | 619.30 | 0.22 | 4.52 | 4.74 | 4.69 |  |  |  |
|  | P5 | 286.08 | 8.02 | 2.55 | 0.08 | 0.10 | 766.7 | 1132.5 | 704.84 | 0.49 | 121.81 | 113.85 | 139.21 |  |  |  |
|  | P95 | 299.99 | 31.40 | 4.13 | 0.59 | 0.57 | 1186.8 | 2394.8 | 4272.05 | 1.65 | 134.89 | 126.06 | 150.77 |  |  |  |
| 2+(1-)^2^+N_10_ | P50 | 413.26 | 10.56 | 3.64 | 0.17 | 0.18 | 860.0 | 1300.9 | 1358.90 | 1.00 | 131.87 | 121.86 | 147.25 | 16 | 491 | 507 |
|  | QD | 38.89 | 0.30 | 0.80 | 0.10 | 0.29 | 52.5 | 198.1 | 386.25 | 0.19 | 3.59 | 4.66 | 4.35 |  |  |  |
|  | P5 | 327.40 | 9.78 | 2.50 | 0.13 | 0.14 | 732.4 | 967.3 | 767.85 | 0.52 | 121.30 | 112.22 | 139.18 |  |  |  |
|  | P95 | 495.25 | 31.05 | 7.17 | 0.59 | 1.83 | 1001.6 | 2127.3 | 3679.46 | 1.56 | 144.55 | 134.76 | 160.13 |  |  |  |
