# Supplementary material for: Diversity of fish sound types in the Pearl River Estuary, China
Source: PeerJ. 2017 Oct 24;5:e3924. doi: 10.7717/peerj.3924 (PMC5659214; doi:10.7717/peerj.3924)
Supplement: Supplemental Information 2 [file peerj-05-3924-s002.zip › Supplemental tables/Supplemental tables/Table S16.docx]

|  |  | Dur | IPPI | τ_95%_ | τ_-3dB_ | τ_-10dB_ | f_p_ | f_c_ | BW_rms_ | Q | SPL_zp_ | SPL_rms_ | EFD | N1 | N2 | N3 |
| --- | --- | --- | --- | --- | --- | --- | --- | --- | --- | --- | --- | --- | --- | --- | --- | --- |
| 2+1+2+N_9_ | P50 | 367.04 | 9.18 | 3.05 | 0.35 | 1.36 | 1180 | 1573 | 737 | 2.06 | 146.30 | 136.85 | 161.22 | 1 | 32 | 33 |
|  | QD | 0.00 | 0.17 | 0.14 | 0.02 | 0.08 | 156 | 22 | 83 | 0.23 | 0.53 | 0.64 | 0.63 |  |  |  |
|  | P5 | 367.04 | 8.04 | 2.43 | 0.20 | 0.91 | 863 | 1496 | 681 | 0.72 | 143.71 | 132.65 | 159.25 |  |  |  |
|  | P95 | 367.04 | 36.62 | 5.48 | 0.39 | 1.57 | 1641 | 2351 | 3057 | 2.31 | 147.73 | 137.83 | 162.54 |  |  |  |
| 2+1+2+N_10_ | P50 | 384.94 | 10.57 | 3.81 | 0.15 | 0.14 | 840 | 1179 | 1337 | 0.92 | 128.31 | 117.69 | 143.06 | 3 | 89 | 92 |
|  | QD | 51.72 | 0.32 | 0.28 | 0.02 | 0.01 | 45 | 94 | 208 | 0.10 | 3.47 | 3.71 | 3.65 |  |  |  |
|  | P5 | 334.10 | 9.72 | 2.77 | 0.11 | 0.11 | 707 | 1034 | 962 | 0.59 | 123.21 | 113.44 | 139.56 |  |  |  |
|  | P95 | 437.54 | 32.45 | 5.00 | 0.22 | 0.16 | 948 | 1429 | 2303 | 1.12 | 133.62 | 123.68 | 149.15 |  |  |  |
