# Supplementary material for: Diversity of fish sound types in the Pearl River Estuary, China
Source: PeerJ. 2017 Oct 24;5:e3924. doi: 10.7717/peerj.3924 (PMC5659214; doi:10.7717/peerj.3924)
Supplement: Supplemental Information 2 [file peerj-05-3924-s002.zip › Supplemental tables/Supplemental tables/Table S17.docx]

|  |  | Dur | IPPI | τ_95%_ | τ_-3dB_ | τ_-10dB_ | f_p_ | f_c_ | BW_rms_ | Q | SPL_zp_ | SPL_rms_ | EFD | N1 | N2 | N3 |
| --- | --- | --- | --- | --- | --- | --- | --- | --- | --- | --- | --- | --- | --- | --- | --- | --- |
| (1-)^4^+N_9_ | P50 | 344.17 | 9.01 | 3.82 | 0.49 | 0.40 | 757.0 | 1092.6 | 1108.90 | 0.95 | 131.57 | 123.48 | 148.77 | 5 | 119 | 124 |
|  | QD | 46.42 | 0.21 | 0.76 | 0.18 | 0.21 | 42.4 | 102.8 | 375.44 | 0.27 | 1.82 | 2.72 | 1.95 |  |  |  |
|  | P5 | 260.85 | 8.35 | 2.83 | 0.17 | 0.18 | 692.3 | 894.4 | 694.33 | 0.50 | 126.54 | 117.69 | 144.37 |  |  |  |
|  | P95 | 368.39 | 47.54 | 6.33 | 1.31 | 1.30 | 907.8 | 1439.0 | 2802.83 | 1.57 | 135.77 | 128.50 | 153.63 |  |  |  |
| (1-)^4^+N_10_ | P50 | 365.45 | 10.53 | 5.79 | 0.14 | 0.16 | 873.5 | 1574.6 | 1641.60 | 1.01 | 129.75 | 117.81 | 144.78 | 22 | 472 | 494 |
|  | QD | 21.38 | 0.27 | 0.90 | 0.02 | 0.27 | 45.1 | 275.0 | 352.58 | 0.14 | 6.23 | 6.61 | 5.97 |  |  |  |
|  | P5 | 169.44 | 9.88 | 3.36 | 0.11 | 0.13 | 696.8 | 1081.0 | 840.37 | 0.66 | 122.12 | 110.43 | 138.62 |  |  |  |
|  | P95 | 451.05 | 46.97 | 7.42 | 0.60 | 1.38 | 1188.0 | 2456.5 | 3462.33 | 1.43 | 146.44 | 133.21 | 159.59 |  |  |  |
| (1-)^4^+N_12_ | P50 | 297.78 | 12.29 | 6.68 | 0.24 | 0.24 | 862.0 | 1346.1 | 1593.50 | 0.86 | 123.43 | 112.35 | 140.26 | 1 | 14 | 15 |
|  | QD | 0.00 | 12.41 | 0.53 | 0.02 | 0.02 | 4.5 | 131.4 | 671.75 | 0.22 | 0.62 | 0.93 | 1.14 |  |  |  |
|  | P5 | 297.78 | 12.05 | 4.08 | 0.03 | 0.04 | 849.0 | 1172.3 | 1059.60 | 0.53 | 120.05 | 108.82 | 137.24 |  |  |  |
|  | P95 | 297.78 | 50.15 | 7.47 | 0.27 | 0.28 | 1058.0 | 2026.5 | 3812.10 | 1.16 | 124.07 | 114.13 | 141.70 |  |  |  |
