# Supplementary material for: Diversity of fish sound types in the Pearl River Estuary, China
Source: PeerJ. 2017 Oct 24;5:e3924. doi: 10.7717/peerj.3924 (PMC5659214; doi:10.7717/peerj.3924)
Supplement: Supplemental Information 2 [file peerj-05-3924-s002.zip › Supplemental tables/Supplemental tables/Table S18.docx]

|  |  | Dur | IPPI | τ_95%_ | τ_-3dB_ | τ_-10dB_ | f_p_ | f_c_ | BW_rms_ | Q | SPL_zp_ | SPL_rms_ | EFD | N1 | N2 | N3 |
| --- | --- | --- | --- | --- | --- | --- | --- | --- | --- | --- | --- | --- | --- | --- | --- | --- |
| (1-)^3^+2+N_10_ | P50 | 383.63 | 10.35 | 6.67 | 0.17 | 0.16 | 1249 | 2088 | 1989 | 1.08 | 121.23 | 108.68 | 136.85 | 4 | 99 | 103 |
|  | QD | 30.38 | 0.32 | 0.56 | 0.02 | 0.02 | 255 | 243 | 286 | 0.17 | 1.85 | 1.64 | 1.40 |  |  |  |
|  | P5 | 361.28 | 9.86 | 5.27 | 0.14 | 0.14 | 829 | 1559 | 1535 | 0.64 | 116.53 | 104.75 | 132.06 |  |  |  |
|  | P95 | 432.94 | 49.83 | 7.28 | 0.22 | 0.24 | 1502 | 2811 | 3122 | 1.28 | 126.33 | 114.14 | 141.44 |  |  |  |
| (1-)^3^+3+N_10_ | P50 | 342.13 | 10.42 | 6.94 | 0.20 | 0.18 | 926 | 1337 | 1552 | 0.87 | 123.56 | 112.81 | 141.08 | 1 | 22 | 23 |
|  | QD | 0.00 | 0.28 | 0.39 | 0.04 | 0.02 | 11 | 60 | 71 | 0.05 | 0.72 | 0.70 | 0.88 |  |  |  |
|  | P5 | 342.13 | 9.97 | 6.01 | 0.06 | 0.06 | 700 | 1181 | 1253 | 0.57 | 119.65 | 109.73 | 137.43 |  |  |  |
|  | P95 | 342.13 | 53.35 | 7.27 | 0.57 | 0.47 | 934 | 1553 | 2730 | 1.01 | 127.10 | 113.87 | 142.09 |  |  |  |
