# Supplementary material for: Diversity of fish sound types in the Pearl River Estuary, China
Source: PeerJ. 2017 Oct 24;5:e3924. doi: 10.7717/peerj.3924 (PMC5659214; doi:10.7717/peerj.3924)
Supplement: Supplemental Information 2 [file peerj-05-3924-s002.zip › Supplemental tables/Supplemental tables/Table S19.docx]

|  |  | Dur | IPPI | τ_95%_ | τ_-3dB_ | τ_-10dB_ | f_p_ | f_c_ | BW_rms_ | Q | SPL_zp_ | SPL_rms_ | EFD | N1 | N2 | N3 |
| --- | --- | --- | --- | --- | --- | --- | --- | --- | --- | --- | --- | --- | --- | --- | --- | --- |
| (1-)^2^+2+1+N_10_ | P50 | 415.86 | 10.23 | 7.35 | 0.20 | 0.18 | 932 | 1297 | 1519 | 0.85 | 124.83 | 112.96 | 141.33 | 1 | 30 | 31 |
|  | QD | 0.00 | 0.15 | 0.40 | 0.03 | 0.01 | 9 | 83 | 334 | 0.12 | 0.70 | 0.68 | 0.65 |  |  |  |
|  | P5 | 415.86 | 9.74 | 6.00 | 0.15 | 0.15 | 914 | 1173 | 1158 | 0.42 | 121.80 | 110.75 | 138.58 |  |  |  |
|  | P95 | 415.86 | 47.60 | 7.78 | 0.45 | 0.24 | 959 | 1761 | 4108 | 1.02 | 127.02 | 114.06 | 142.58 |  |  |  |
| (1-)^2^+2+3+N_10_ | P50 | 314.28 | 10.65 | 5.69 | 0.13 | 0.14 | 923 | 1914 | 2284 | 0.83 | 119.20 | 107.55 | 135.37 | 1 | 20 | 21 |
|  | QD | 0.00 | 0.97 | 0.30 | 0.01 | 0.01 | 10 | 283 | 589 | 0.09 | 1.43 | 1.12 | 1.08 |  |  |  |
|  | P5 | 314.28 | 9.88 | 5.28 | 0.08 | 0.07 | 884 | 1522 | 1888 | 0.50 | 116.45 | 105.31 | 132.93 |  |  |  |
|  | P95 | 314.28 | 47.51 | 6.81 | 0.17 | 0.18 | 950 | 3691 | 7074 | 0.93 | 120.69 | 109.73 | 136.87 |  |  |  |
