# Supplementary material for: Diversity of fish sound types in the Pearl River Estuary, China
Source: PeerJ. 2017 Oct 24;5:e3924. doi: 10.7717/peerj.3924 (PMC5659214; doi:10.7717/peerj.3924)
Supplement: Supplemental Information 2 [file peerj-05-3924-s002.zip › Supplemental tables/Supplemental tables/Table S2.docx]

|  |  | Dur | IPPI | τ95% | τ-3dB | τ-10dB | fp | fc | BWrms | Q | SPLzp | SPLrms | EFD | N1 | N2 | N3 |
| --- | --- | --- | --- | --- | --- | --- | --- | --- | --- | --- | --- | --- | --- | --- | --- | --- |
| 2+N_9_ | P50 | 299.88 | 9.08 | 3.28 | 0.59 | 1.03 | 809 | 1121 | 1329 | 0.77 | 127.21 | 120.42 | 144.85 | 5 | 137 | 142 |
|  | QD | 24.40 | 0.24 | 0.83 | 0.27 | 0.50 | 52 | 120 | 814 | 0.34 | 2.88 | 2.82 | 2.83 |  |  |  |
|  | P5 | 253.49 | 8.34 | 2.48 | 0.05 | 0.19 | 734 | 911 | 612 | 0.42 | 122.12 | 112.24 | 140.14 |  |  |  |
|  | P95 | 324.42 | 10.41 | 7.10 | 1.11 | 2.14 | 937 | 3137 | 5667 | 1.74 | 133.88 | 126.66 | 150.69 |  |  |  |
| 2+N_10_ | P50 | 304.54 | 10.28 | 3.06 | 0.20 | 0.20 | 830 | 1099 | 1045 | 1.03 | 130.32 | 121.14 | 145.75 | 4 | 101 | 105 |
|  | QD | 39.30 | 0.19 | 0.54 | 0.06 | 0.17 | 44 | 135 | 216 | 0.12 | 3.62 | 3.02 | 3.87 |  |  |  |
|  | P5 | 233.03 | 9.53 | 2.56 | 0.15 | 0.15 | 744 | 1000 | 892 | 0.62 | 127.45 | 118.59 | 143.73 |  |  |  |
|  | P95 | 331.08 | 12.14 | 5.69 | 0.47 | 1.51 | 1098 | 1554 | 2540 | 1.24 | 142.64 | 135.60 | 160.04 |  |  |  |
| 2+N_18_ | P50 | 171.10 | 17.79 | 4.60 | 0.53 | 1.02 | 796 | 1106 | 503 | 2.26 | 158.05 | 149.65 | 175.71 | 42 | 295 | 337 |
|  | QD | 11.94 | 1.51 | 0.53 | 0.07 | 0.46 | 51 | 98 | 38 | 0.25 | 3.98 | 3.47 | 3.57 |  |  |  |
|  | P5 | 133.13 | 13.39 | 3.74 | 0.47 | 0.90 | 707 | 863 | 407 | 1.09 | 142.63 | 133.82 | 162.13 |  |  |  |
|  | P95 | 214.20 | 65.53 | 7.34 | 0.83 | 3.17 | 953 | 1251 | 834 | 2.66 | 161.75 | 152.90 | 179.42 |  |  |  |
