# Supplementary material for: Diversity of fish sound types in the Pearl River Estuary, China
Source: PeerJ. 2017 Oct 24;5:e3924. doi: 10.7717/peerj.3924 (PMC5659214; doi:10.7717/peerj.3924)
Supplement: Supplemental Information 2 [file peerj-05-3924-s002.zip › Supplemental tables/Supplemental tables/Table S20.docx]

|  |  | Dur | IPPI | τ_95%_ | τ_-3dB_ | τ_-10dB_ | f_p_ | f_c_ | BW_rms_ | Q | SPL_zp_ | SPL_rms_ | EFD | N1 | N2 | N3 |
| --- | --- | --- | --- | --- | --- | --- | --- | --- | --- | --- | --- | --- | --- | --- | --- | --- |
| 2+(1-)^3^+N_10_ | P50 | 401.47 | 10.47 | 5.84 | 0.17 | 0.17 | 845 | 1407 | 1734 | 0.84 | 127.41 | 116.32 | 143.36 | 7 | 214 | 221 |
|  | QD | 17.27 | 0.22 | 0.97 | 0.04 | 0.03 | 41 | 241 | 459 | 0.15 | 1.97 | 1.74 | 1.72 |  |  |  |
|  | P5 | 364.65 | 9.79 | 3.90 | 0.11 | 0.13 | 747 | 1093 | 1059 | 0.47 | 123.22 | 112.38 | 139.96 |  |  |  |
|  | P95 | 448.94 | 29.85 | 7.42 | 0.44 | 0.24 | 980 | 2556 | 4411 | 1.11 | 130.84 | 120.00 | 146.33 |  |  |  |
| 2+(1-)^4^+N_10_ | P50 | 394.93 | 10.59 | 5.73 | 0.17 | 0.17 | 764 | 1452 | 1482 | 0.97 | 122.87 | 112.71 | 140.24 | 1 | 27 | 28 |
|  | QD | 0.00 | 0.18 | 0.19 | 0.01 | 0.01 | 103 | 86 | 177 | 0.07 | 1.51 | 0.91 | 0.97 |  |  |  |
|  | P5 | 394.93 | 10.21 | 5.33 | 0.15 | 0.15 | 713 | 1253 | 1259 | 0.59 | 118.57 | 109.77 | 136.93 |  |  |  |
|  | P95 | 394.93 | 44.48 | 6.75 | 0.23 | 0.20 | 1048 | 1918 | 3377 | 1.09 | 124.81 | 114.92 | 142.17 |  |  |  |
