# Supplementary material for: Diversity of fish sound types in the Pearl River Estuary, China
Source: PeerJ. 2017 Oct 24;5:e3924. doi: 10.7717/peerj.3924 (PMC5659214; doi:10.7717/peerj.3924)
Supplement: Supplemental Information 2 [file peerj-05-3924-s002.zip › Supplemental tables/Supplemental tables/Table S21.docx]

|  |  | Dur | IPPI | τ_95%_ | τ_-3dB_ | τ_-10dB_ | f_p_ | f_c_ | BW_rms_ | Q | SPL_zp_ | SPL_rms_ | EFD | N1 | N2 | N3 |
| --- | --- | --- | --- | --- | --- | --- | --- | --- | --- | --- | --- | --- | --- | --- | --- | --- |
| (1-)^5^+N_9_ | P50 | 340.30 | 9.10 | 3.11 | 0.27 | 0.24 | 916 | 1699 | 1167 | 1.26 | 138.19 | 129.73 | 154.36 | 2 | 46 | 48 |
|  | QD | 26.52 | 0.35 | 0.31 | 0.15 | 0.18 | 206 | 254 | 559 | 0.55 | 8.41 | 7.44 | 7.94 |  |  |  |
|  | P5 | 313.78 | 8.43 | 2.23 | 0.04 | 0.04 | 735 | 1033 | 894 | 0.42 | 121.50 | 114.79 | 138.53 |  |  |  |
|  | P95 | 366.81 | 48.82 | 3.89 | 1.29 | 1.12 | 1898 | 2412 | 5618 | 1.93 | 141.86 | 132.64 | 157.95 |  |  |  |
| (1-)^5^+N_10_ | P50 | 387.27 | 10.63 | 6.19 | 0.14 | 0.14 | 878 | 1707 | 1780 | 0.97 | 128.38 | 116.11 | 143.53 | 23 | 526 | 549 |
|  | QD | 22.81 | 0.34 | 1.20 | 0.02 | 0.02 | 47 | 359 | 287 | 0.12 | 2.54 | 2.23 | 2.35 |  |  |  |
|  | P5 | 256.63 | 10.02 | 3.09 | 0.11 | 0.11 | 674 | 967 | 1014 | 0.63 | 122.09 | 110.58 | 136.85 |  |  |  |
|  | P95 | 425.75 | 46.30 | 7.50 | 0.30 | 1.02 | 1499 | 2843 | 3355 | 1.43 | 132.93 | 122.19 | 148.66 |  |  |  |
