# Supplementary material for: Diversity of fish sound types in the Pearl River Estuary, China
Source: PeerJ. 2017 Oct 24;5:e3924. doi: 10.7717/peerj.3924 (PMC5659214; doi:10.7717/peerj.3924)
Supplement: Supplemental Information 2 [file peerj-05-3924-s002.zip › Supplemental tables/Supplemental tables/Table S22.docx]

|  |  | Dur | IPPI | τ95% | τ-3dB | τ-10dB | fp | fc | BWrms | Q | SPLzp | SPLrms | EFD | N1 | N2 | N3 |
| --- | --- | --- | --- | --- | --- | --- | --- | --- | --- | --- | --- | --- | --- | --- | --- | --- |
| (1-)^4^+2+N_10_ | P50 | 405.78 | 10.70 | 6.38 | 0.16 | 0.16 | 937 | 1876 | 1905 | 1.02 | 124.59 | 112.45 | 140.42 | 8 | 193 | 201 |
|  | QD | 35.76 | 0.26 | 0.45 | 0.01 | 0.02 | 161 | 298 | 318 | 0.13 | 3.56 | 3.41 | 3.57 |  |  |  |
|  | P5 | 314.32 | 9.97 | 4.85 | 0.13 | 0.13 | 764 | 1438 | 1478 | 0.61 | 119.19 | 107.38 | 134.76 |  |  |  |
|  | P95 | 478.90 | 48.74 | 7.46 | 0.21 | 0.25 | 1582 | 2811 | 4287 | 1.28 | 131.75 | 118.81 | 146.43 |  |  |  |
| (1-)^4^+3+N_11_ | P50 | 342.45 | 11.28 | 6.10 | 0.18 | 0.22 | 2162 | 2695 | 1851 | 1.42 | 132.15 | 119.17 | 146.63 | 1 | 17 | 18 |
|  | QD | 0.00 | 5.87 | 0.38 | 0.02 | 0.03 | 603 | 285 | 253 | 0.09 | 2.08 | 1.59 | 1.51 |  |  |  |
|  | P5 | 342.45 | 10.67 | 2.78 | 0.15 | 0.16 | 820 | 2283 | 1525 | 0.93 | 125.57 | 114.45 | 142.55 |  |  |  |
|  | P95 | 342.45 | 80.79 | 6.73 | 0.38 | 0.36 | 2357 | 3708 | 3628 | 1.65 | 134.10 | 122.38 | 148.56 |  |  |  |
