# Supplementary material for: Diversity of fish sound types in the Pearl River Estuary, China
Source: PeerJ. 2017 Oct 24;5:e3924. doi: 10.7717/peerj.3924 (PMC5659214; doi:10.7717/peerj.3924)
Supplement: Supplemental Information 2 [file peerj-05-3924-s002.zip › Supplemental tables/Supplemental tables/Table S23.docx]

|  |  | Dur | IPPI | τ_95%_ | τ_-3dB_ | τ_-10dB_ | f_p_ | f_c_ | BW_rms_ | Q | SPL_zp_ | SPL_rms_ | EFD | N1 | N2 | N3 |
| --- | --- | --- | --- | --- | --- | --- | --- | --- | --- | --- | --- | --- | --- | --- | --- | --- |
| (1-)^3^+2+1+N_10_ | P50 | 358.48 | 10.68 | 6.12 | 0.14 | 0.14 | 832 | 2296 | 2133 | 1.03 | 124.74 | 112.62 | 140.58 | 4 | 88 | 92 |
|  | QD | 22.38 | 0.36 | 0.29 | 0.02 | 0.02 | 107 | 517 | 344 | 0.25 | 2.10 | 1.70 | 1.88 |  |  |  |
|  | P5 | 347.87 | 10.12 | 4.79 | 0.04 | 0.04 | 550 | 737 | 1402 | 0.44 | 118.04 | 108.26 | 135.29 |  |  |  |
|  | P95 | 400.48 | 57.11 | 6.74 | 1.24 | 1.37 | 979 | 4050 | 5967 | 1.23 | 130.38 | 118.19 | 145.88 |  |  |  |
| (1-)^4^+2+1+N_10_ | P50 | 404.97 | 10.47 | 6.20 | 0.65 | 1.89 | 755 | 1030 | 1195 | 0.87 | 123.82 | 113.72 | 141.74 | 1 | 22 | 23 |
|  | QD | 0.00 | 4.53 | 0.39 | 0.23 | 0.08 | 7 | 90 | 138 | 0.05 | 1.12 | 0.59 | 0.65 |  |  |  |
|  | P5 | 404.97 | 9.65 | 5.95 | 0.23 | 1.73 | 737 | 918 | 1017 | 0.68 | 120.07 | 110.72 | 138.37 |  |  |  |
|  | P95 | 404.97 | 64.97 | 6.97 | 1.03 | 2.30 | 766 | 1248 | 1643 | 1.02 | 125.42 | 114.80 | 142.58 |  |  |  |
