# Supplementary material for: Diversity of fish sound types in the Pearl River Estuary, China
Source: PeerJ. 2017 Oct 24;5:e3924. doi: 10.7717/peerj.3924 (PMC5659214; doi:10.7717/peerj.3924)
Supplement: Supplemental Information 2 [file peerj-05-3924-s002.zip › Supplemental tables/Supplemental tables/Table S24.docx]

|  |  | Dur | IPPI | τ_95%_ | τ_-3dB_ | τ_-10dB_ | f_p_ | f_c_ | BW_rms_ | Q | SPL_zp_ | SPL_rms_ | EFD | N1 | N2 | N3 |
| --- | --- | --- | --- | --- | --- | --- | --- | --- | --- | --- | --- | --- | --- | --- | --- | --- |
| (1-)^6^+N_10_ | P50 | 380.72 | 10.74 | 6.28 | 0.17 | 0.18 | 924 | 2048 | 1852 | 0.97 | 126.30 | 113.93 | 141.43 | 8 | 179 | 187 |
|  | QD | 39.37 | 3.02 | 0.63 | 0.03 | 0.03 | 197 | 749 | 531 | 0.18 | 1.54 | 1.89 | 1.46 |  |  |  |
|  | P5 | 337.82 | 10.09 | 3.07 | 0.13 | 0.13 | 718 | 1027 | 1124 | 0.54 | 122.38 | 110.10 | 137.49 |  |  |  |
|  | P95 | 453.66 | 52.85 | 7.95 | 0.27 | 0.60 | 2638 | 3208 | 4118 | 1.45 | 131.88 | 119.78 | 148.59 |  |  |  |
| (1-)^7^+N_10_ | P50 | 371.85 | 10.88 | 6.49 | 0.15 | 0.15 | 879 | 1613 | 1847 | 0.86 | 124.24 | 112.45 | 140.41 | 5 | 108 | 113 |
|  | QD | 46.84 | 5.46 | 0.50 | 0.02 | 0.02 | 58 | 231 | 473 | 0.14 | 3.82 | 3.92 | 3.87 |  |  |  |
|  | P5 | 336.94 | 10.02 | 4.50 | 0.11 | 0.11 | 778 | 1278 | 1457 | 0.51 | 119.64 | 109.17 | 136.34 |  |  |  |
|  | P95 | 445.23 | 48.71 | 7.39 | 0.23 | 0.92 | 1049 | 2533 | 4537 | 1.09 | 137.73 | 124.81 | 151.84 |  |  |  |
