# Supplementary material for: Diversity of fish sound types in the Pearl River Estuary, China
Source: PeerJ. 2017 Oct 24;5:e3924. doi: 10.7717/peerj.3924 (PMC5659214; doi:10.7717/peerj.3924)
Supplement: Supplemental Information 2 [file peerj-05-3924-s002.zip › Supplemental tables/Supplemental tables/Table S25.docx]

|  |  | Dur | IPPI | τ_95%_ | τ_-3dB_ | τ_-10dB_ | f_p_ | f_c_ | BW_rms_ | Q | SPL_zp_ | SPL_rms_ | EFD | N1 | N2 | N3 |
| --- | --- | --- | --- | --- | --- | --- | --- | --- | --- | --- | --- | --- | --- | --- | --- | --- |
| (1-)^5^+2+N_10_ | P50 | 428.69 | 10.77 | 6.30 | 0.16 | 0.17 | 866 | 1658 | 1784 | 0.94 | 123.08 | 112.66 | 140.40 | 3 | 69 | 72 |
|  | QD | 31.40 | 3.33 | 1.11 | 0.06 | 0.05 | 256 | 623 | 478 | 0.11 | 2.63 | 3.51 | 3.74 |  |  |  |
|  | P5 | 378.41 | 10.34 | 3.49 | 0.11 | 0.11 | 457 | 816 | 865 | 0.60 | 117.22 | 105.21 | 132.82 |  |  |  |
|  | P95 | 441.21 | 60.55 | 7.36 | 1.01 | 0.33 | 1320 | 2616 | 3631 | 1.28 | 126.39 | 116.18 | 143.95 |  |  |  |
| (1-)^5^+3+N_10_ | P50 | 408.70 | 10.64 | 7.59 | 0.16 | 0.17 | 1241 | 2567 | 1928 | 1.28 | 128.88 | 115.69 | 144.25 | 1 | 23 | 24 |
|  | QD | 0.00 | 4.84 | 0.27 | 0.02 | 0.06 | 284 | 215 | 347 | 0.15 | 1.14 | 1.04 | 1.02 |  |  |  |
|  | P5 | 408.70 | 9.97 | 7.04 | 0.05 | 0.06 | 672 | 2059 | 1643 | 0.69 | 124.81 | 113.40 | 141.80 |  |  |  |
|  | P95 | 408.70 | 63.06 | 7.76 | 0.29 | 0.31 | 2392 | 4533 | 6309 | 1.45 | 130.88 | 116.94 | 145.63 |  |  |  |
|  |  |  |  |  |  |  |  |  |  |  |  |  |  |  |  |  |
