# Supplementary material for: Diversity of fish sound types in the Pearl River Estuary, China
Source: PeerJ. 2017 Oct 24;5:e3924. doi: 10.7717/peerj.3924 (PMC5659214; doi:10.7717/peerj.3924)
Supplement: Supplemental Information 2 [file peerj-05-3924-s002.zip › Supplemental tables/Supplemental tables/Table S26.docx]

|  |  | Dur | IPPI | τ95% | τ-3dB | τ-10dB | fp | fc | BWrms | Q | SPLzp | SPLrms | EFD | N1 | N2 | N3 |
| --- | --- | --- | --- | --- | --- | --- | --- | --- | --- | --- | --- | --- | --- | --- | --- | --- |
| (1-)^4^+(2-)^2^+N_10_ | P50 | 439.23 | 10.41 | 5.11 | 0.18 | 0.16 | 909 | 1281 | 1301.30 | 0.97 | 125.67 | 114.72 | 141.63 | 1 | 27 | 28 |
|  | QD | 0.00 | 0.28 | 0.52 | 0.03 | 0.01 | 66 | 41 | 135.35 | 0.08 | 1.02 | 0.60 | 0.72 |  |  |  |
|  | P5 | 439.23 | 9.90 | 4.18 | 0.15 | 0.14 | 829 | 1191 | 1114.68 | 0.51 | 122.85 | 113.27 | 140.00 |  |  |  |
|  | P95 | 439.23 | 51.82 | 6.03 | 0.30 | 0.20 | 1097 | 1558 | 3050.52 | 1.11 | 128.57 | 116.97 | 143.83 |  |  |  |
| (1-)^5^+(2-)^2^+N_10_ | P50 | 399.47 | 11.28 | 6.52 | 1.14 | 0.86 | 728 | 986 | 1724.50 | 0.73 | 123.52 | 113.85 | 141.69 | 2 | 40 | 42 |
|  | QD | 62.71 | 6.05 | 0.43 | 0.57 | 0.61 | 86 | 591 | 487.39 | 0.16 | 1.92 | 0.71 | 1.35 |  |  |  |
|  | P5 | 336.76 | 9.90 | 4.08 | 0.04 | 0.05 | 571 | 733 | 946.29 | 0.37 | 120.32 | 111.25 | 138.97 |  |  |  |
|  | P95 | 462.18 | 56.86 | 7.15 | 1.45 | 1.60 | 970 | 2813 | 5253.96 | 1.13 | 129.56 | 116.32 | 144.39 |  |  |  |
