# Supplementary material for: Diversity of fish sound types in the Pearl River Estuary, China
Source: PeerJ. 2017 Oct 24;5:e3924. doi: 10.7717/peerj.3924 (PMC5659214; doi:10.7717/peerj.3924)
Supplement: Supplemental Information 2 [file peerj-05-3924-s002.zip › Supplemental tables/Supplemental tables/Table S3.docx]

|  |  | Dur | IPPI | τ_95%_ | τ_-3dB_ | τ_-10dB_ | f_p_ | f_c_ | BW_rms_ | Q | SPL_zp_ | SPL_rms_ | EFD | N1 | N2 | N3 |
| --- | --- | --- | --- | --- | --- | --- | --- | --- | --- | --- | --- | --- | --- | --- | --- | --- |
| 3+N_9_ | P50 | 276.08 | 9.13 | 3.69 | 0.28 | 0.22 | 1041 | 1581 | 1835 | 0.85 | 125.73 | 116.48 | 141.95 | 8 | 228 | 236 |
|  | QD | 15.13 | 0.28 | 0.26 | 0.10 | 0.11 | 143 | 187 | 824 | 0.25 | 4.25 | 3.53 | 3.59 |  |  |  |
|  | P5 | 259.23 | 8.27 | 2.83 | 0.06 | 0.11 | 825 | 1146 | 792 | 0.47 | 122.38 | 114.09 | 139.46 |  |  |  |
|  | P95 | 335.51 | 9.78 | 5.06 | 0.65 | 1.85 | 1301 | 2816 | 5358 | 1.73 | 135.08 | 126.43 | 152.79 |  |  |  |
| 3+N_10_ | P50 | 167.97 | 10.80 | 6.22 | 0.17 | 0.72 | 889 | 1735 | 2247 | 0.86 | 123.61 | 111.27 | 138.39 | 42 | 741 | 783 |
|  | QD | 110.04 | 0.29 | 1.06 | 0.04 | 0.51 | 432 | 815 | 818 | 0.21 | 4.12 | 4.31 | 4.47 |  |  |  |
|  | P5 | 126.63 | 9.95 | 2.76 | 0.06 | 0.13 | 651 | 977 | 931 | 0.47 | 116.47 | 103.04 | 130.55 |  |  |  |
|  | P95 | 473.94 | 37.92 | 8.37 | 0.65 | 1.82 | 2529 | 4452 | 5584 | 1.49 | 134.72 | 123.09 | 149.87 |  |  |  |
| 3+N_17_ | P50 | 168.13 | 17.24 | 5.21 | 0.79 | 0.82 | 779 | 878 | 557 | 1.59 | 141.75 | 134.01 | 161.33 | 17 | 133 | 150 |
|  | QD | 11.09 | 1.78 | 1.17 | 0.10 | 0.19 | 55 | 96 | 193 | 0.54 | 5.60 | 5.58 | 4.85 |  |  |  |
|  | P5 | 143.60 | 12.70 | 3.68 | 0.33 | 0.55 | 601 | 713 | 342 | 0.60 | 132.65 | 125.46 | 152.71 |  |  |  |
|  | P95 | 200.23 | 51.46 | 7.36 | 1.23 | 3.70 | 889 | 1183 | 1752 | 2.44 | 161.13 | 152.74 | 178.77 |  |  |  |
