# Supplementary material for: Diversity of fish sound types in the Pearl River Estuary, China
Source: PeerJ. 2017 Oct 24;5:e3924. doi: 10.7717/peerj.3924 (PMC5659214; doi:10.7717/peerj.3924)
Supplement: Supplemental Information 2 [file peerj-05-3924-s002.zip › Supplemental tables/Supplemental tables/Table S4.docx]

|  |  | Dur | IPPI | τ_95%_ | τ_-3dB_ | τ_-10dB_ | f_p_ | f_c_ | BW_rms_ | Q | SPL_zp_ | SPL_rms_ | EFD | N1 | N2 | N3 |
| --- | --- | --- | --- | --- | --- | --- | --- | --- | --- | --- | --- | --- | --- | --- | --- | --- |
| 4+N_9_ | P50 | 278.07 | 9.15 | 4.64 | 0.31 | 0.23 | 991 | 1538 | 2032 | 0.75 | 125.08 | 115.17 | 141.85 | 5 | 149 | 154 |
|  | QD | 24.08 | 0.24 | 1.16 | 0.10 | 0.33 | 152 | 228 | 1009 | 0.29 | 3.66 | 1.67 | 1.47 |  |  |  |
|  | P5 | 269.24 | 8.32 | 3.08 | 0.04 | 0.06 | 736 | 1298 | 796 | 0.47 | 122.32 | 112.34 | 139.53 |  |  |  |
|  | P95 | 337.97 | 9.66 | 6.65 | 0.68 | 1.63 | 1245 | 3127 | 5723 | 1.91 | 142.23 | 132.58 | 157.34 |  |  |  |
| 4+N_10_ | P50 | 215.83 | 10.60 | 5.05 | 0.17 | 0.18 | 914 | 1858 | 1765 | 0.99 | 126.59 | 114.45 | 141.62 | 17 | 355 | 372 |
|  | QD | 129.10 | 0.23 | 1.25 | 0.07 | 0.21 | 207 | 518 | 624 | 0.18 | 3.13 | 4.71 | 4.12 |  |  |  |
|  | P5 | 120.16 | 9.79 | 2.97 | 0.10 | 0.10 | 594 | 734 | 629 | 0.54 | 117.20 | 104.03 | 131.43 |  |  |  |
|  | P95 | 435.76 | 14.36 | 7.35 | 1.32 | 1.83 | 2596 | 3635 | 4938 | 1.50 | 135.62 | 125.46 | 152.08 |  |  |  |
| 4+N_17_ | P50 | 177.76 | 16.87 | 6.85 | 0.72 | 0.73 | 663 | 787 | 737 | 1.08 | 138.69 | 130.35 | 158.55 | 1 | 9 | 10 |
|  | QD | 0.00 | 2.10 | 0.17 | 0.14 | 0.15 | 3 | 37 | 66 | 0.05 | 0.48 | 0.86 | 0.84 |  |  |  |
|  | P5 | 177.76 | 12.13 | 5.73 | 0.48 | 0.01 | 660 | 749 | 520 | 0.62 | 136.25 | 128.90 | 156.30 |  |  |  |
|  | P95 | 177.76 | 41.17 | 6.97 | 0.89 | 0.91 | 758 | 965 | 1565 | 1.47 | 139.85 | 131.31 | 159.55 |  |  |  |
