# Supplementary material for: Diversity of fish sound types in the Pearl River Estuary, China
Source: PeerJ. 2017 Oct 24;5:e3924. doi: 10.7717/peerj.3924 (PMC5659214; doi:10.7717/peerj.3924)
Supplement: Supplemental Information 2 [file peerj-05-3924-s002.zip › Supplemental tables/Supplemental tables/Table S5.docx]

|  |  | Dur | IPPI | τ_95%_ | τ_-3dB_ | τ_-10dB_ | f_p_ | f_c_ | BW_rms_ | Q | SPL_zp_ | SPL_rms_ | EFD | N1 | N2 | N3 |
| --- | --- | --- | --- | --- | --- | --- | --- | --- | --- | --- | --- | --- | --- | --- | --- | --- |
| 5+N_10_ | P50 | 141.28 | 10.82 | 6.67 | 0.20 | 0.23 | 2400 | 3091 | 2773 | 1.14 | 118.81 | 105.73 | 133.79 | 5 | 44 | 49 |
|  | QD | 16.03 | 0.39 | 0.30 | 0.05 | 0.05 | 502 | 262 | 618 | 0.23 | 3.32 | 2.65 | 2.56 |  |  |  |
|  | P5 | 129.58 | 10.08 | 5.33 | 0.09 | 0.11 | 523 | 2059 | 2043 | 0.51 | 112.45 | 100.06 | 128.17 |  |  |  |
|  | P95 | 172.29 | 57.40 | 7.39 | 0.33 | 0.35 | 2585 | 4218 | 6180 | 1.43 | 123.20 | 109.67 | 137.78 |  |  |  |
