# Supplementary material for: Diversity of fish sound types in the Pearl River Estuary, China
Source: PeerJ. 2017 Oct 24;5:e3924. doi: 10.7717/peerj.3924 (PMC5659214; doi:10.7717/peerj.3924)
Supplement: Supplemental Information 2 [file peerj-05-3924-s002.zip › Supplemental tables/Supplemental tables/Table S6.docx]

|  |  | Dur | IPPI | τ_95%_ | τ_-3dB_ | τ_-10dB_ | f_p_ | f_c_ | BW_rms_ | Q | SPL_zp_ | SPL_rms_ | EFD | N1 | N2 | N3 |
| --- | --- | --- | --- | --- | --- | --- | --- | --- | --- | --- | --- | --- | --- | --- | --- | --- |
| (1-)^2^+N_9_ | P50 | 269.28 | 8.98 | 3.15 | 0.35 | 0.36 | 837 | 1247 | 1653 | 0.67 | 125.68 | 119.06 | 143.38 | 3 | 67 | 70 |
|  | QD | 18.66 | 0.18 | 0.41 | 0.16 | 0.18 | 35 | 147 | 793 | 0.31 | 6.64 | 6.20 | 5.91 |  |  |  |
|  | P5 | 236.80 | 8.41 | 2.44 | 0.04 | 0.04 | 676 | 934 | 693 | 0.41 | 121.08 | 111.89 | 137.48 |  |  |  |
|  | P95 | 274.11 | 34.05 | 4.37 | 0.91 | 1.21 | 1067 | 2524 | 4808 | 1.90 | 138.38 | 129.79 | 154.24 |  |  |  |
| (1-)^2^+N_10_ | P50 | 226.74 | 10.21 | 3.60 | 0.41 | 1.29 | 929 | 1407 | 744 | 1.88 | 150.13 | 139.85 | 165.46 | 49 | 859 | 908 |
|  | QD | 33.85 | 0.25 | 0.63 | 0.04 | 0.16 | 153 | 105 | 156 | 0.37 | 3.69 | 3.97 | 3.55 |  |  |  |
|  | P5 | 156.92 | 9.84 | 2.35 | 0.16 | 0.38 | 800 | 1121 | 548 | 0.86 | 133.87 | 125.82 | 150.95 |  |  |  |
|  | P95 | 340.26 | 33.18 | 6.66 | 0.72 | 1.67 | 1365 | 1824 | 2140 | 2.56 | 157.03 | 146.87 | 171.80 |  |  |  |
| (1-)^2^+N_12_ | P50 | 197.51 | 12.03 | 3.91 | 0.38 | 0.36 | 872 | 1277 | 1076 | 1.15 | 133.78 | 125.33 | 151.13 | 23 | 295 | 318 |
|  | QD | 33.94 | 0.48 | 0.62 | 0.11 | 0.11 | 31 | 137 | 416 | 0.48 | 9.46 | 10.05 | 9.67 |  |  |  |
|  | P5 | 119.96 | 11.47 | 2.72 | 0.17 | 0.18 | 774 | 985 | 584 | 0.56 | 120.88 | 112.35 | 138.44 |  |  |  |
|  | P95 | 309.06 | 38.61 | 5.06 | 0.92 | 1.15 | 964 | 1858 | 3213 | 2.31 | 148.94 | 140.00 | 164.41 |  |  |  |
