# Supplementary material for: Diversity of fish sound types in the Pearl River Estuary, China
Source: PeerJ. 2017 Oct 24;5:e3924. doi: 10.7717/peerj.3924 (PMC5659214; doi:10.7717/peerj.3924)
Supplement: Supplemental Information 2 [file peerj-05-3924-s002.zip › Supplemental tables/Supplemental tables/Table S7.docx]

|  |  | Dur | IPPI | τ_95%_ | τ_-3dB_ | τ_-10dB_ | f_p_ | f_c_ | BW_rms_ | Q | SPL_zp_ | SPL_rms_ | EFD | N1 | N2 | N3 |
| --- | --- | --- | --- | --- | --- | --- | --- | --- | --- | --- | --- | --- | --- | --- | --- | --- |
| 1+2+N_10_ | P50 | 232.96 | 10.08 | 3.78 | 0.45 | 0.47 | 948 | 1284 | 652 | 1.96 | 146.11 | 136.48 | 162.13 | 1 | 18 | 19 |
|  | QD | 0.00 | 0.24 | 0.38 | 0.02 | 0.01 | 33 | 15 | 142 | 0.33 | 0.29 | 0.48 | 0.24 |  |  |  |
|  | P5 | 232.96 | 10.01 | 2.75 | 0.40 | 0.43 | 840 | 1252 | 562 | 1.06 | 142.01 | 132.77 | 158.81 |  |  |  |
|  | P95 | 232.96 | 32.75 | 4.60 | 0.91 | 0.50 | 992 | 1419 | 1340 | 2.30 | 146.65 | 138.41 | 162.75 |  |  |  |
| 1+2+N_18_ | P50 | 294.46 | 18.30 | 7.76 | 0.80 | 0.81 | 666 | 822 | 695 | 1.14 | 135.38 | 127.85 | 156.405 | 2 | 16 | 18 |
|  | QD | 25.80 | 20.01 | 0.16 | 0.06 | 0.05 | 43 | 58 | 88 | 0.12 | 1.98 | 1.13 | 1.46 |  |  |  |
|  | P5 | 268.66 | 13.67 | 5.81 | 0.05 | 0.11 | 594 | 676 | 495 | 0.42 | 133.16 | 124.43 | 153.21 |  |  |  |
|  | P95 | 320.25 | 108.15 | 8.15 | 1.63 | 1.39 | 798 | 1030 | 2466 | 1.63 | 138.55 | 129.78 | 158.44 |  |  |  |
