# Supplementary material for: Diversity of fish sound types in the Pearl River Estuary, China
Source: PeerJ. 2017 Oct 24;5:e3924. doi: 10.7717/peerj.3924 (PMC5659214; doi:10.7717/peerj.3924)
Supplement: Supplemental Information 2 [file peerj-05-3924-s002.zip › Supplemental tables/Supplemental tables/Table S8.docx]

|  |  | Dur | IPPI | τ_95%_ | τ_-3dB_ | τ_-10dB_ | f_p_ | f_c_ | BW_rms_ | Q | SPL_zp_ | SPL_rms_ | EFD | N1 | N2 | N3 |
| --- | --- | --- | --- | --- | --- | --- | --- | --- | --- | --- | --- | --- | --- | --- | --- | --- |
| 2+1+N_9_ | P50 | 298.02 | 9.15 | 3.38 | 0.40 | 0.41 | 901 | 1472 | 1236 | 1.05 | 129.93 | 121.60 | 146.82 | 12 | 337 | 349 |
|  | QD | 29.23 | 0.22 | 0.36 | 0.08 | 0.09 | 176 | 139 | 469 | 0.37 | 3.39 | 2.70 | 2.45 |  |  |  |
|  | P5 | 247.01 | 8.31 | 2.58 | 0.16 | 0.15 | 729 | 889 | 698 | 0.51 | 122.95 | 114.75 | 140.19 |  |  |  |
|  | P95 | 378.53 | 25.37 | 4.27 | 0.77 | 1.38 | 1214 | 2045 | 3693 | 1.97 | 138.03 | 129.11 | 154.32 |  |  |  |
| 2+1+N_10_ | P50 | 385.33 | 10.47 | 3.88 | 0.19 | 0.61 | 836 | 1208 | 1178 | 1.15 | 134.58 | 125.95 | 151.70 | 11 | 352 | 363 |
|  | QD | 38.09 | 0.24 | 0.95 | 0.09 | 0.71 | 49 | 170 | 412 | 0.24 | 3.51 | 4.51 | 4.60 |  |  |  |
|  | P5 | 346.00 | 9.75 | 2.65 | 0.11 | 0.11 | 775 | 906 | 625 | 0.55 | 123.92 | 113.85 | 139.40 |  |  |  |
|  | P95 | 493.59 | 29.11 | 6.17 | 0.92 | 1.90 | 976 | 2173 | 3068 | 1.58 | 143.13 | 129.73 | 156.41 |  |  |  |
