# Supplementary material for: Diversity of fish sound types in the Pearl River Estuary, China
Source: PeerJ. 2017 Oct 24;5:e3924. doi: 10.7717/peerj.3924 (PMC5659214; doi:10.7717/peerj.3924)
Supplement: Supplemental Information 2 [file peerj-05-3924-s002.zip › Supplemental tables/Supplemental tables/Table S9.docx]

|  |  | Dur | IPPI | τ_95%_ | τ_-3dB_ | τ_-10dB_ | f_p_ | f_c_ | BW_rms_ | Q | SPL_zp_ | SPL_rms_ | EFD | N1 | N2 | N3 |
| --- | --- | --- | --- | --- | --- | --- | --- | --- | --- | --- | --- | --- | --- | --- | --- | --- |
| (2-)^2^+N_10_ | P50 | 363.57 | 10.53 | 6.20 | 0.18 | 0.55 | 992 | 1898 | 2146 | 0.90 | 122.16 | 110.91 | 138.87 | 3 | 84 | 87 |
|  | QD | 22.32 | 0.22 | 0.67 | 0.09 | 0.72 | 270 | 409 | 781 | 0.32 | 2.30 | 1.34 | 1.01 |  |  |  |
|  | P5 | 332.39 | 9.87 | 3.91 | 0.09 | 0.11 | 644 | 944 | 1505 | 0.29 | 119.59 | 108.80 | 136.45 |  |  |  |
|  | P95 | 377.03 | 28.38 | 7.36 | 1.16 | 2.18 | 1518 | 3192 | 6489 | 1.20 | 127.01 | 114.84 | 141.15 |  |  |  |
| 4+1+N_10_ | P50 | 406.74 | 10.62 | 5.40 | 0.15 | 0.15 | 886 | 1613 | 1977 | 0.79 | 122.71 | 110.96 | 138.57 | 6 | 199 | 205 |
|  | QD | 43.09 | 0.20 | 0.83 | 0.02 | 0.01 | 49 | 279 | 396 | 0.13 | 3.41 | 3.02 | 3.11 |  |  |  |
|  | P5 | 290.52 | 9.79 | 3.61 | 0.11 | 0.11 | 789 | 1205 | 1208 | 0.53 | 118.58 | 108.01 | 134.73 |  |  |  |
|  | P95 | 432.41 | 23.67 | 7.38 | 0.25 | 0.18 | 1109 | 2962 | 4321 | 1.08 | 131.25 | 120.61 | 147.15 |  |  |  |
