# Supplementary material for: Diversity of fish sound types in the Pearl River Estuary, China
Source: PeerJ. 2017 Oct 24;5:e3924. doi: 10.7717/peerj.3924 (PMC5659214; doi:10.7717/peerj.3924)
Supplement: Supplemental Information 2 [file peerj-05-3924-s002.zip › Supplemental tables/Supplemental Table legends.docx]

**Table S1 Descriptive statistics of the sonic parameters of single and paired pulse call types.**

P50, median; P5 and P95, 5th percentile and 95th percentile, respectively; QD, quartile deviation; Dur, duration; IPPI, inter-pulsepeak interval; τ_95%_, duration of 95% cumulative energy; τ_-3dB_ andτ_-10dB_, duration of -3 dB and -10 dB of the peak amplitude of the enveloped signal, respectively; f_p_, peak frequency; f_c_, center frequency; BW_rms_, centralized root-mean-square bandwidth; Q, quality factor; SPL_zp_ and SPL_rms_, zero-to-peak and root-mean-square sound pressure levels, respectively; EFD, energy flux density; N1, N2 and N3, number of calls, inter-pulsepeak intervals and pulses analyzed, respectively. The duration is in seconds, the frequency is in Hz, the SPL is in dB re 1 µPa, and the EFD is in dB re 1µPa^2^s. The IPIs are not shown here and can be obtained by subtracting 8 ms from the IPPIs. The same notation was used for the following tables.

**Table S2 Descriptive statistics of sonic parameters of the 2+N_9_, 2+N_10_ and 2+N_18_ call types.**

**Table S3 Descriptive statistics of sonic parameters of the 3+N_9_, 3+N_10_ and 3+N_17_ call types.**

**Table S4 Descriptive statistics of sonic parameters of the 4+N_9_, 4+N_10_ and 4+N_17_ call types.**

**Table S5 Descriptive statistics of sonic parameters of 5+N_10_ call type.**

**Table S6 Descriptive statistics of sonic parameters of the (1-)^2^+N_9_, (1-)^2^+N_10_ and (1-)^2^+N_12_ call types.**

**Table S7 Descriptive statistics of sonic parameters of the 1+2+N_10_ and 1+2+N_18_ call types.**

**Table S8 Descriptive statistics of sonic parameters of the 2+1+N_9_ and 2+1+N_10_ call types.**

**Table S9 Descriptive statistics of sonic parameters of the (2-)^2^+N_10_ and 4+1+N_10_ call types.**

**Table S10 Descriptive statistics of sonic parameters of the 3+1+N_9_ and 3+1+N_10_ call types.**

**Table S11 Descriptive statistics of sonic parameters of the 3+2+N_9_ and 3+(1-)^2^+N_9_ call types.**

**Table S12 Descriptive statistics of sonic parameters of the (1-)^3^+N_9_, (1-)^3^+N_10_ and (1-)^3^+N_12_ call types.**

**Table S13 Descriptive statistics of sonic parameters of the (1-)^2^+2+N_9_ and (1-)^2^+2+N_10_ call types.**

**Table S14 Descriptive statistics of sonic parameters of the (1-)^2^+3+N_10_ call type.**

**Table S15 Descriptive statistics of sonic parameters of the 2+(1-)^2^+N_9_ and 2+(1-)^2^+N_10_ call types.**

**Table S16 Descriptive statistics of sonic parameters of the 2+1+2+N_9_ and 2+1+2+N_10_ call types.**

**Table S17 Descriptive statistics of sonic parameters of the (1-)^4^+N_9_, (1-)^4^+N_10_ and (1-)^4^+N_12_ call types.**

**Table S18 Descriptive statistics of sonic parameters of the (1-)^3^+2+N_10_ and (1-)^3^+3+N_10_ call types.**

**Table S19 Descriptive statistics of sonic parameters of the (1-)^2^+2+1+N_10_ and (1-)^2^+2+3+N_10_ call types.**

**Table S20 Descriptive statistics of sonic parameters of the 2+(1-)^3^+N_10_ and 2+(1-)^4^+N_10_ call types.**

**Table S21 Descriptive statistics of sonic parameters of the (1-)^5^+N_9_ and (1-)^5^+N_10_ call types.**

**Table S22 Descriptive statistics of sonic parameters of the (1-)^4^+2+N_10_ and (1-)^4^+3+N_11_ call types.**

**Table S23 Descriptive statistics of sonic parameters of the (1-)^3^+2+1+N_10_ and (1-)^4^+2+1+N_10_ call types.**

**Table S24 Descriptive statistics of sonic parameters of the (1-)^6^+N_10_ and (1-)^7^+N_10_ call types.**

**Table S25 Descriptive statistics of sonic parameters of the (1-)^5^+2+N_10_ and (1-)^5^+3+N_10_ call types.**

**Table S26 Descriptive statistics of sonic parameters of the (1-)^4^+(2-)^2^+N_10_ and (1-)^5^+(2-)^2^+N_10_ call types.**
